# The Dynamic Alliance of p53 and Metabolism in the Tumor Microenvironment Shapes Tumor Evolution

**DOI:** 10.1002/bies.70175

**Published:** 2026-08-19

**Authors:** Sebastien M. Joruiz, Francesco Napoletano, Rebecca Bertolio, Giannino Del Sal

**Affiliations:** ^1^ International Centre for Genetic Engineering and Biotechnology (ICGEB) Area Science Park‐Padriciano Trieste Italy; ^2^ Department of Life Sciences University of Trieste Trieste Italy; ^3^ IFOM ETS the AIRC Institute of Molecular Oncology Milan Italy

**Keywords:** cell competition, metabolic plasticity, metastasis, mutant p53, p53, p53 isoforms, tumor microenvironment

## Abstract

Tumor evolution, from premalignant lesions to metastasis, is increasingly recognized as shaped by continuous interplay between tumor cells metabolism and their microenvironment. During tumor initiation, major oncogenic pathways drive early metabolic reprogramming of lipid, amino acid, and energy pathways to promote cell competition and clonal expansion. These metabolic changes reciprocally shape the tumor microenvironment (TME) through metabolite fluxes, extracellular matrix remodeling, and immune reprogramming, generating adaptive niches that sustain tumor progression and metastasis. Cancer cell metabolic adaptability becomes even more crucial to survive dissemination and adapt to a new, distant microenvironment.

Here, we discuss these dynamic interplays and highlight the p53 pathway as an integrative hub linking oncogenic signaling, metabolic rewiring, and tumor microenvironmental adaptation throughout carcinogenesis. We will also outline how emerging technologies may redefine the TME‐p53‐metabolism interplay uncovering therapeutically exploitable metabolic vulnerabilities.

## Graphical abstract

p53 is an adaptive processor positioned at the interface between metabolism and the tumor microenvironment. By integrating nutrient, mechanical, inflammatory, and redox cues while reciprocally remodeling metabolism, stromal and immune compartments, and cancer cell plasticity, WT and mutant p53 orchestrate cell competition, tumor evolution, metastasis, and therapy response.

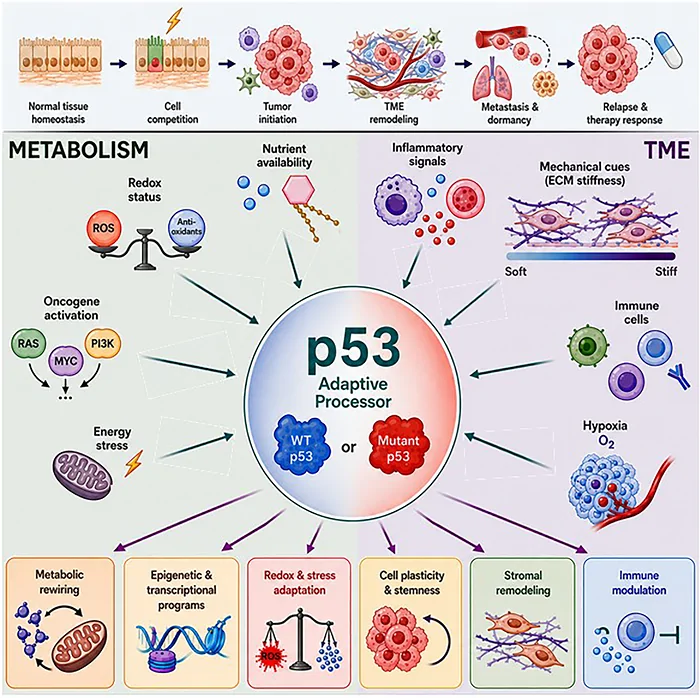

## 1 Introduction

Metabolic alterations, a cancer hallmark, support malignant transformation throughout tumorigenesis [1]. Cancer cells emerging in permissive conditions (e.g., aging, inflamed, or fibrotic tissues) face nutrient fluctuations, oxidative stress, and altered extracellular matrix (ECM) architecture [2]. To survive, they adopt metabolic programs that favor rapid ATP generation, biosynthetic flexibility, and redox balance. As tumors progress, these adaptations enable cancer cells to withstand hypoxia, fuel rapid proliferation, and accumulate biomass. During metastasis, metabolic plasticity allows to survive in circulation, invade foreign tissues, and adapt to distinct metabolic landscapes [2]. This plasticity also contributes to therapy resistance by enabling dynamic shift between metabolic modes in response to chemo‐, targeted, and immune therapy [3].

A major driver of this metabolic rewiring is oncogenic signaling. For example, mutant RAS enhances glucose uptake, glycolysis, glutamine dependence, and lipid synthesis [4]. Similarly, PI3K/AKT/mTOR signaling increases nutrient uptake and macromolecular synthesis, while suppressing catabolic pathways such as autophagy [5]. These oncogenic signals converge on metabolic nodes that maintain redox homeostasis and survival. They also facilitate metastasis by supporting membrane synthesis (e.g., fatty acids, cholesterol), cytoskeletal remodeling, and secretion of factors that prepare pre‐metastatic niches (e.g., cytokines, metalloproteinases, and ECM components) [6, 7].

The metabolism of cancer cells is intertwined with their dynamic interaction with the tumor microenvironment (TME) [8, 9]. Cancer cells actively remodel the ECM, immune landscape, and stromal compartment, fostering tumor progression, immune evasion, and treatment resistance [1, 3, 6, 7, 10, 11], and in turn stromal, immune, and vascular cells influence tumor metabolism [1, 11, 12]. This bidirectional relationship includes mechano‐metabolic circuits, where tumor metabolism affects the TME physical properties, while mechanical cues regulate membranes, enzymes, organelles, and metabolic gene programs [11]. Spatial differences in oxygen and nutrient availability further generates metabolic heterogeneity, increasing tumor adaptability, aggressiveness and treatment resistance [13]. Furthermore, systemic factors such as diet and metabolic diseases influence organ‐specific metabolic differences contributing to tumorigenesis [14, 15].

This complex interplay highlights the importance of regulatory hubs like the p53 pathway [16], which integrates oncogenic signals, stress responses and extracellular cues. Known as a tumor‐suppressor, p53 is a transcription factor that orchestrates cellular responses to stress, by coordinating cell‐cycle arrest, genome stability, senescence and apoptosis. The human TP53 gene encodes twelve isoforms, including the full‐length p53 protein (p53α), which collectively regulate the pathway activity [17]. The conservation of both TP53 sequence and isoforms‐generating mechanisms across Metazoa underscores their fundamental biological functions [17, 18, 19, 20].

Beyond tumor‐suppression, p53 is a major regulator of metabolism and tissue homeostasis [16]. It regulates glycolysis, lipid metabolism, oxidative phosphorylation (OXPHOS), antioxidant defense, autophagy, and ferroptosis, thereby, counteracting oncogene‐driven metabolic programs [16, 21, 22]. Furthermore, wild type (WT) p53α directly interacts with oncogenic pathways, including MAPK and PI3K/AKT/mTOR signaling, acting as a metabolic sensor and regulator [23, 24]. Other p53 isoforms also contribute to sensing and regulating metabolic cues, by fine‐tuning p53α activity and exerting independent functions in response to metabolic stresses (e.g., hypoxia, nutrient shortage), influencing mitochondrial activity and TME remodeling [24, 25, 26, 27, 28]. Alterations of the p53 pathway are among the most frequent events in multiple types of cancer, with prevalence of TP53 missense mutations that hamper tumor‐suppressive activity and can confer gain of oncogenic functions (GOF) [29]. About 92% of TP53 mutations affect all p53 isoforms, and mutant p53 isoforms also contribute to carcinogenesis [30]. Mutant p53 proteins lose DNA binding capacity; however, they interact with transcription factors and chromatin modifiers, and reshape gene expression, metabolism and microenvironmental interactions, favoring tumor development [29, 31]. Examples include WNT activation through PI3K/AKT [32], suppression of cGAS/STING/Interferon‐I (IFN‐I) innate immunity signaling [33], and mechanotransduction through the mutant p53/mevalonate/RhoA axis, which in turn activates YAP/TAZ [34, 35]. In contrast to WT p53, which inhibits the mevalonate pathway [36], mutant p53, thus acts as a mechano‐activated oncogene controlling key metabolic pathways.

Hence, p53 stands at the crossroads of metabolic regulation, oncogenic signaling, and TME adaptation. In this review, we analyze the complex interplay between these processes and highlight how they are integrated and modulated by the p53 pathway to sustain tumor initiation, progression, metastasis, and resistance to therapy. We also discuss approaches to study this dynamic interplay and identify therapeutic vulnerabilities.

## 2 Interplay Between Metabolism and Oncogenes in Tumor Initiation

### 2.1 Cell Competition and Metabolic Fitness in Premalignant Tissues

**FIGURE 1:**
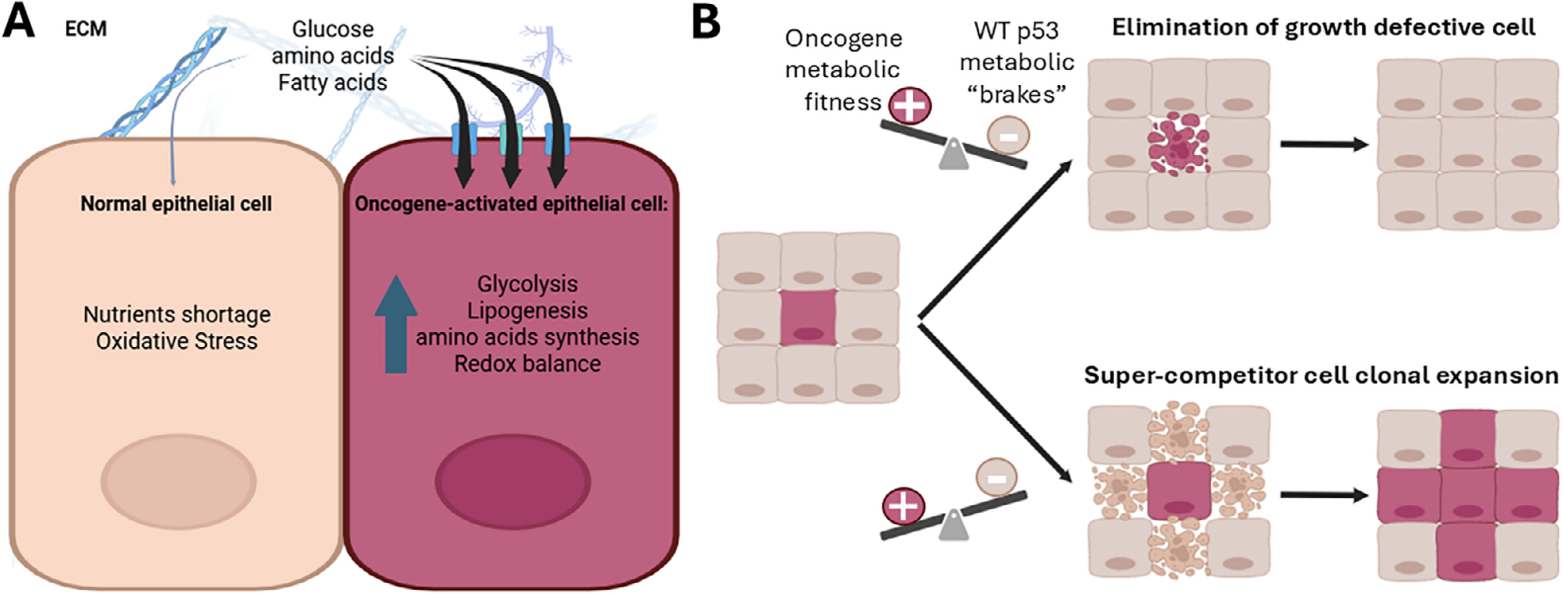
Metabolic fitness and cell competition during tumor initiation. Early tumor evolution is shaped by competition between cells with distinct metabolic capacities. (**A**) Oncogenic activation of PI3K/AKT/mTOR, RAS/RAF/ERK, YAP/TAZ pathways or p53 mutation confers a metabolic fitness advantage. (B) Metabolic fitness is balanced by metabolic brakes triggered by WT p53. If metabolic brakes prevail, the growth‐defective cell is eliminated. However, if metabolic fitness prevails, this metabolically optimized cell outcompetes surrounding cells and clonally expands.

Tumors develop through genetic and epigenetic alterations that occur in cells over time and are selected by tissue microenvironmental alterations, allowing survival, and expansion of best‐adapted cell clones. Recent genome sequencing studies have unveiled that cell clones carrying cancer‐driver mutations (TP53, PI3K, RAS, NOTCH) accumulate with age in many tissues well before observable histological alterations, and the fitness of these cells is critically dependent on the tissue microenvironment [37]. These cells engage interactions and compete for nutrients and space with the surrounding WT cells, and multiple evidence have shown that the resulting clonal dynamics is tightly linked with the regulation of cell metabolism in response to microenvironmental cues and with the status of the p53 pathway activation [38] (Figure 1).

In healthy tissues, cells harboring cancer‐driving mutations are held in check by normal neighbors through cell competition, a process conserved from *Drosophila* to mammals, in which cells with different fitness interact by ligand/receptor, secreted factors, and/or mechanically [39]. The fittest cells emerge within the subpopulation as winners, while loser counterparts undergo either death, senescence, engulfment, extrusion, or differentiation. For example, cells expressing missense mutant forms of p53, one of the most frequently mutated genes in both normal tissues and cancer, display metabolic alterations and are frequently outcompeted in mouse tissues (e.g., intestine, pancreas) and human cell cultures [40, 41]. Also, WT epithelial cells can recognize and actively eliminate cells in which oncogenic mutant RAS stimulates PDK4‐dependent Warburg‐effect‐like metabolic rewiring [42]. Moreover, SCRIBBLE knockout pre‐neoplastic cells or cells with reduced mitochondrial activity and anabolic capacity typically express elevated WT p53 levels. This hypersensitizes them to p53 inducing stress (e.g., mechanical stress) and lead them to be outcompeted in a WT p53‐dependent manner [43, 44, 45, 46].

While emerging pre‐neoplastic cells can be eliminated by cell competition, which physiologically functions in normal tissues as a tumor suppressive mechanism, accumulating evidence has shown that aging‐associated microenvironmental alterations known to increase cancer risk, such as metabolic dysfunctions, inflammation and fibrosis, favor the competitive expansion of cells harboring cancer‐driving mutations (e.g., expressing oncogenic mutant p53, MYC, YAP). A paradigmatic case is represented by clones of missense mutant p53 cells, which expand with age in different tissues, including esophagus, skin, blood, colon, and breast [47]. Multiple evidences suggest that in this process a key role is played by mutant p53‐dependent metabolic rewiring. For example, in recent studies in elegant mouse models of breast and liver cancer driven by the induction of monoallelic missense mutations in p53 in sporadic cells, tumor initiation and progression have been shown to involve mutant p53‐dependent rewiring of glycolysis, OXPHOS. NADH, reactive oxygen species (ROS), and lipid metabolism in response to genotoxic and metabolic perturbations (e.g., high fat diet) [15, 48, 49, 50, 51, 52] (Table 1). Furthermore, in mouse models of mutant p53‐driven cancer, the use of genetic reporters of mutant p53 protein levels has allowed to establish that in different tumors, including lymphoma, sarcoma, and carcinoma, the increase of mutant p53 levels and activity marks precancerous clones and promotes amino acid uptake and metabolism to support tumor‐initiating capacity [53]. In addition, works in mouse models showed that, in hematopoietic and esophageal stem/progenitor cells experiencing genotoxic and mitochondrial stress, p53 mutation promotes mutant cell stress adaptation and clonal expansion at the expense of WT neighsboring cells [54, 55].

**TABLE 1:** Differential regulation of metabolic and microenvironmental adaptation by WT and mutant p53 during tumor evolution.

Biological process	WT p53	Mutant p53	Representative mechanisms	Ref.
Glycolysis	Suppresses glycolysis through TIGAR and metabolic checkpoints; limits glucose uptake and glycolytic overflow	Enhances glycolysis through HIF‐1α cooperation, PDK1 induction and increased glucose utilization, supporting clonal expansion and tumor progression	TIGAR, PDK1, HIF‐1α	[22, 29, 49, 50, 51, 52, 53, 54, 56, 57, 58]
Oxidative phosphorylation / mitochondrial metabolism	Promotes mitochondrial respiration and oxidative metabolism, mtDNA maintenance and metabolic efficiency	Rewires mitochondrial metabolism to sustain anabolic growth and metastatic adaptation; enhances mitochondrial fitness during tumor initiation	SCO2, POLγ, mitochondrial biogenesis, ROS buffering	[6, 22, 26, 29, 53, 54, 59, 60, 61]
Lipid metabolism	Restrains lipogenesis and maintains lipid homeostasis	Promotes fatty acid synthesis, cholesterol biosynthesis, mevalonate pathway and peroxisomal metabolism to support proliferation and metastasis	SREBP/mevalonate axis; cholesterol metabolism	[13, 29, 35, 36, 49, 50, 51, 52, 59, 62]
Amino acid metabolism	Coordinates serine, glycine and one‐carbon metabolism; adapts to nutrient deprivation via AMPK/ISR and adaptive amino acid utilization	Enhances amino acid uptake (LAT1/CD98), serine synthesis pathway and glutamine metabolism under nutrient stress	LAT1/CD98, SSP enzymes, one‐carbon metabolism	[29, 49, 50, 51, 52, 63, 64]
Redox homeostasis	Maintains physiological ROS through antioxidant and metabolic programs; enforces arrest/apoptosis when oxidative stress becomes excessive	Activates NRF2‐dependent antioxidant programs, glutathione metabolism and NADPH production, allowing tolerance to oxidative stress and therapy resistance	TIGAR, NRF2, glutathione metabolism, ROS buffering	[22, 29, 31, 49, 50, 51, 52, 53, 54, 56, 57, 60, 61]
mTOR signaling / anabolic metabolism	Restrains mTORC1 activity through DDIT4, RFX7, PTEN, TSC1, and related targets	Activates mTORC1‐dependent anabolic metabolism in response to metabolic and genotoxic stress, increasing metabolic fitness	mTORC1; AKT; nutrient sensing	[14, 23, 24, 29, 48, 49, 50, 51, 52, 57]
Extracellular matrix remodeling & mechanotransduction	Regulates ECM organization and mechanosensing; Δ133 isoforms regulate laminins and fibronectin; Δ40 promotes collagen III in fibrosis	Promotes ECM stiffening, MMP expression, TIMP3 repression, HIF‐1α/miR‐30d secretome, mevalonate‐RhoA‐YAP signaling	MMPs, TIMP3, miR‐30d, RhoA, YAP/TAZ	[6, 11, 19, 27, 28, 29, 35, 36, 65, 66]
Immune regulation / immune evasion	Restrains inflammatory signaling; activates cGAS/STING; modulates SASP; limits chronic NF‐κB/STAT3 activation	Cooperates with NF‐κB to sustain inflammation, promotes immune suppression, IDO1 induction, impaired antigen presentation and T‐cell dysfunction	cGAS/STING, TREX1, IDO1, NF‐κB	[6, 7, 27, 29, 30, 31, 33, 67, 68, 69, 70, 71]
Response to nutrient deprivation & hypoxia	Activates AMPK, inhibits mTOR, induces metabolic adaptation or apoptosis; integrates HIF signaling	Stabilized by nutrient stress; promotes LAT1, serine synthesis, PDK1 and metabolic adaptation	AMPK, mTOR, LAT1, PDK1	[24, 25, 26, 57, 58, 64, 72]
Cancer cell plasticity	Can promote quiescence, senescence or dormancy depending on context	Promotes stemness, EMT, invasion and metastatic plasticity	YAP/TAZ, inflammatory transcription factors, metabolic rewiring	[17, 19, 29, 30, 31, 35, 73, 74, 75]
Metastatic adaptation	Generally restrains dissemination through metabolic checkpoints, although Δ133 isoforms can promote migration and invasion	Enhances dissemination, organ colonization, metastatic niche remodeling, oxidative stress tolerance and metastatic outgrowth	Brain metastasis, HIF‐1α, PDK1, antioxidant pathways	[6, 17, 19, 30, 58, 59, 60, 61, 62, 76, 77, 78, 79]
**SUMMARY**				
Adaptive processor of the tumor ecosystem	Integrates metabolic and microenvironmental stress to preserve tissue homeostasis, restrain oncogenic metabolism and coordinate adaptive responses	Rewires the same sensing pathways to promote metabolic flexibility, immune evasion, ECM remodeling and metastatic fitness	Nutrient sensing, mechanotransduction, inflammatory signaling, metabolic PTMs	

Insights from Li‐Fraumeni syndrome (LFS) further underscore the role of p53 expression and mutation status in shaping metabolic states during tumor initiation. LFS is an autosomal dominant genetic condition in which patients present a germline mutation in the TP53 gene, leading to early onset tumors, particularly sarcoma, breast cancer, leukemia, and adrenal gland cancer. In these patients, p53 loss of heterozygosity arises years before tumor diagnosis, implicating it as an early driver of precancer evolution in LFS. It induces subtle, but systemic metabolic rewiring, including altered lipid utilization, amino acid metabolism, and oxidative stress responses [80, 81]. These changes generate a permissive landscape for tumor initiation upon a new genetic event, such as oncogene activation, gradual accumulation of mutations or a tissue injury inducing proliferation pathways [82]. Whether, other p53 isoforms are directly involved in cell competition has not been investigated, but evidence clearly suggests that they probably are. For example, synonymous TP53 mutation leads to RNA secondary structure formation favoring Δ40p53α expression, and is associated with glioblastoma and ovarian cancer development [82]. Similarly, a mutation specifically affecting the STOP codon of the β isoforms, while the other isoforms, including canonical p53α, are WT, gives rise to an early onset autosomal dominant cancer syndrome with colorectal, breast, and papillary thyroid cancers [83].

While cell competition in premalignant tissues is fundamentally shaped by intrinsic metabolic fitness differences between p53 mutant and WT clones, oncogenic signaling pathways further amplify these differences by actively reprogramming cellular metabolism to confer competitive dominance.

### 2.2 Oncogenic Metabolic Programs and the Gatekeeping Role of WT p53

Besides mutant p53, other oncogenic signaling pathways such as PI3K/AKT/mTOR, RAS/ERK and YAP/TAZ actively reprogram cellular metabolism to enhance clonal fitness and promote competitive expansion within premalignant tissues. In this context, WT p53 acts as a key counterbalance that restrains oncogene‐driven metabolic rewiring and preserves tissue homeostasis (Figure 1).

For example, it was recently demonstrated that the PI3K/AKT/mTOR signaling confers clonal dominance to nascent esophageal cancer cells in response to organismal supraphysiological insulin levels [14]. It was also reported that AKT‐hyperactivated cells exhibit increased glucose and amino acid consumption, metabolically disadvantaging adjacent normal cells and facilitating clonal expansion during early tumorigenesis [84, 85]. Similarly, KRAS‐mutant cells exhibit increased nutrient scavenging, including macropinocytosis and elevated glucose and amino acid consumption, depriving neighboring normal cells, and enabling clonal expansion during early tumorigenesis [86]. Moreover, ERK‐driven metabolic rewiring can induce non–cell‐autonomous effects by altering local nutrient availability and redox balance, reinforcing competitive dominance within premalignant tissues [87, 88]. The YAP/TAZ pathway also acts on metabolism to induce cell competition. Indeed, activation of Yorkie/YAP promotes super‐competitor behavior through induction of Myc‐dependent growth and metabolic programs that increase cellular fitness during competition [89, 90]. Similar Hippo‐TEAD‐Myc fitness competition mechanisms have been described in mammalian cells [91]. This supports a conserved role for Hippo signaling in metabolic determinants of cellular fitness, thereby promoting cellular fitness and the emergence of super‐competitor cells during tissue competition [92]. Altogether, these oncogenes commonly reprogram nascent cancer cell metabolism, including glucose, fatty acids, and amino acids, to promote clonal expansion.

Nevertheless, WT p53 plays a crucial role in these oncogene‐driven growth‐promoting programs. Of note, the following p53 putative functions described in cancer cells may apply in the cell competition context. For example, WT p53α suppresses aerobic glycolysis by transcriptionally inducing TIGAR and promoting redox control [22]. Similarly, it transcriptionally regulates genes involved in mitochondrial respiration and biogenesis, promoting ATP production over glycolytic overflow [59]. WT Δ40p53α also participates in this regulation by upregulating TIGAR, p21 and 14‐3‐3σ expression [24], and by interacting with p53α to stimulate the mitochondrial DNA pol‐γ [26]. Through these functions, the p53 pathway constrains the biosynthetic and metabolic capacity promoted by oncogenic pathways. Furthermore, p53 directly interacts with these oncogenic pathways to directly tamper them. For example, it was recently shown that p53α inhibits AKT and mTOR in a DDIT4‐ and RFX7‐dependent manner, which also depends on nutrient availability. In this context, p53‐RFX7‐mediated mTORC1 inhibits nutrient‐associated mTORC1 activity, keeping metabolic activity in check despite nutrient excess conditions. This underlines the context‐dependent role of the p53 pathway acting as a crucial metabolic sensor and regulator [23]. It also transcriptionally upregulates genes such as PTEN, ASS1, PHLDA1/3, or PLK2/TSC1, which are direct inhibitors of AKT and mTOR [93]. Similarly, recent evidence demonstrates that p53 and MAPK signaling regulate each other and collaborate to determine cell fate, particularly the balance between cell proliferation and cell death [56]. In this context, stress‐activated kinases such as ERK, JNK, and p38 modulate p53 dynamics, while p53 transcriptional programs restrain MAPK‐driven proliferation [56].

All these pieces of evidence highlight that the tumor suppressor p53 occupies a unique position at the intersection of metabolic regulation and cell competition during tumor initiation, functioning as both a metabolic gatekeeper in its WT form and a metabolic rewiring factor when mutated. Through both its WT tumor‐suppressive functions and mutant GOF, the p53 pathway integrates metabolic control with cell competition, shaping clonal selection and tissue ecology during tumor initiation in direct counterpoint or cooperation with PI3K/AKT/mTOR, YAP/TAZ, and RAS/RAF/ERK signaling networks.

## 3 Metabolic Rewiring in the TME During Tumor Progression

### 3.1 The Tumor Metabolism and the Microenvironment Dynamic Ecosystem

As tumors progress, cancer cell metabolism becomes increasingly intertwined with the changing TME, and oncogene‐driven metabolic programs contribute to the remodeling of the surrounding stroma, immune landscape, and ECM. Importantly, these interactions are bidirectional: the metabolic state of stromal and immune cells not only responds to tumor‐derived signals, but also feeds back to regulate p53 activity, stability and transcriptional programs within cancer cells, establishing dynamic adaptive circuits that drive tumor progression and therapy resistance [1, 8, 10].

Pro‐oncogenic metabolic rewiring, including enhanced glycolysis, glutamine utilization and lipid biosynthesis, all of which are regulated by p53 as discussed above, generates metabolites that actively remodel the TME. For instance, through its down‐regulation of MCT1 expression, WT p53 is a major inhibitor of lactate secretion, while p53 loss leads to lactate accumulation in the TME [65]. Lactate accumulation acidifies the extracellular space, promoting matrix remodeling, angiogenesis, and activation of cancer‐associated fibroblasts (CAFs) and tumor‐associated macrophages (TAMs) toward tumor‐supportive phenotypes [1, 94, 95]. CAF‐driven ECM deposition and crosslinking increase tissue stiffness, activating integrin signaling and YAP/TAZ‐dependent mechanotransduction, which reinforces glycolysis, lipid metabolism and mitochondrial function, establishing a reinforcing feedback loop between metabolic and biomechanical cues [11, 96].

Interestingly, p53 status strongly influences this metabolic symbiosis. WT p53 restrains glycolytic flux, promotes oxidative phosphorylation and limits oxidative stress, thereby reducing lactate production and diminishing the metabolic pressure that drives CAF glycolysis. Conversely, mutant p53 enhances glycolysis, lactate export, and anabolic metabolism. In response, CAF undergo autophagy and glycolysis and will secrete lactate, pyruvate and amino acids that fuel oxidative metabolism in cancer cells, a phenomenon dubbed “reverse Warburg effect” [97]. The impact of this metabolic symbiosis in tumor progression is underlined by observations that high stromal MCT4 expression, a lactate and pyruvate transporter, correlates with poor prognosis and enhanced tumor growth [98]. Reciprocal lipid exchange also occurs as adipocytes and CAFs can provide fatty acids and phospholipids to cancer cells, while tumor‐derived signals enhance lipolysis and lipid remodeling in stromal compartments, reinforcing energy availability and membrane synthesis, creating a self‐reinforcing metabolic circuit that favors tumor progression [99, 100, 101].

Metabolic rewiring also shapes immune suppression within the TME. Nutrient depletion and lactate accumulation impair effector T cell and natural killer (NK) cell function, partly through epigenetic mechanisms such as histone lactylation [102]. ECM remodeling further limits immune infiltration and function, while YAP/TAZ activation in stromal cells reinforces immune exclusion [11, 103]. Beyond responding to tumor‐derived metabolites, stromal, and immune cells actively reshape p53 signaling through reciprocal metabolic interactions. CAF‐derived metabolites modify nutrient availability and cellular redox balance, while inflammatory cytokines, hypoxia, and mechanical cues collectively influence the functional state of the p53 pathway, determining whether cancer cells adapt arrest or acquire tumor‐promoting phenotypes [1, 3, 16, 29, 57].

Likewise, inflammatory cytokines produced by TAMs, T cells and endothelial cells activate NF‐κB, STAT3 and MAPK signaling, whereas chronic hypoxia and nutrient deprivation modulate HIF‐1α‐p53 crosstalk [12, 104, 105], collectively modulating p53‐dependent stress responses in cancer cells. Endothelial cells also participate in this metabolic reciprocity. In response to hypoxia, endothelial metabolic reprogramming drives angiogenesis and dynamically regulates oxygen and nutrient delivery, while endothelial‐derived cytokines, angiogenic growth factors and metabolic signals reshape tumor metabolism and immune‐cell recruitment, thereby indirectly influencing HIF‐1α–p53 signaling and the adaptive response of tumor cells [57, 106].

**FIGURE 2:**
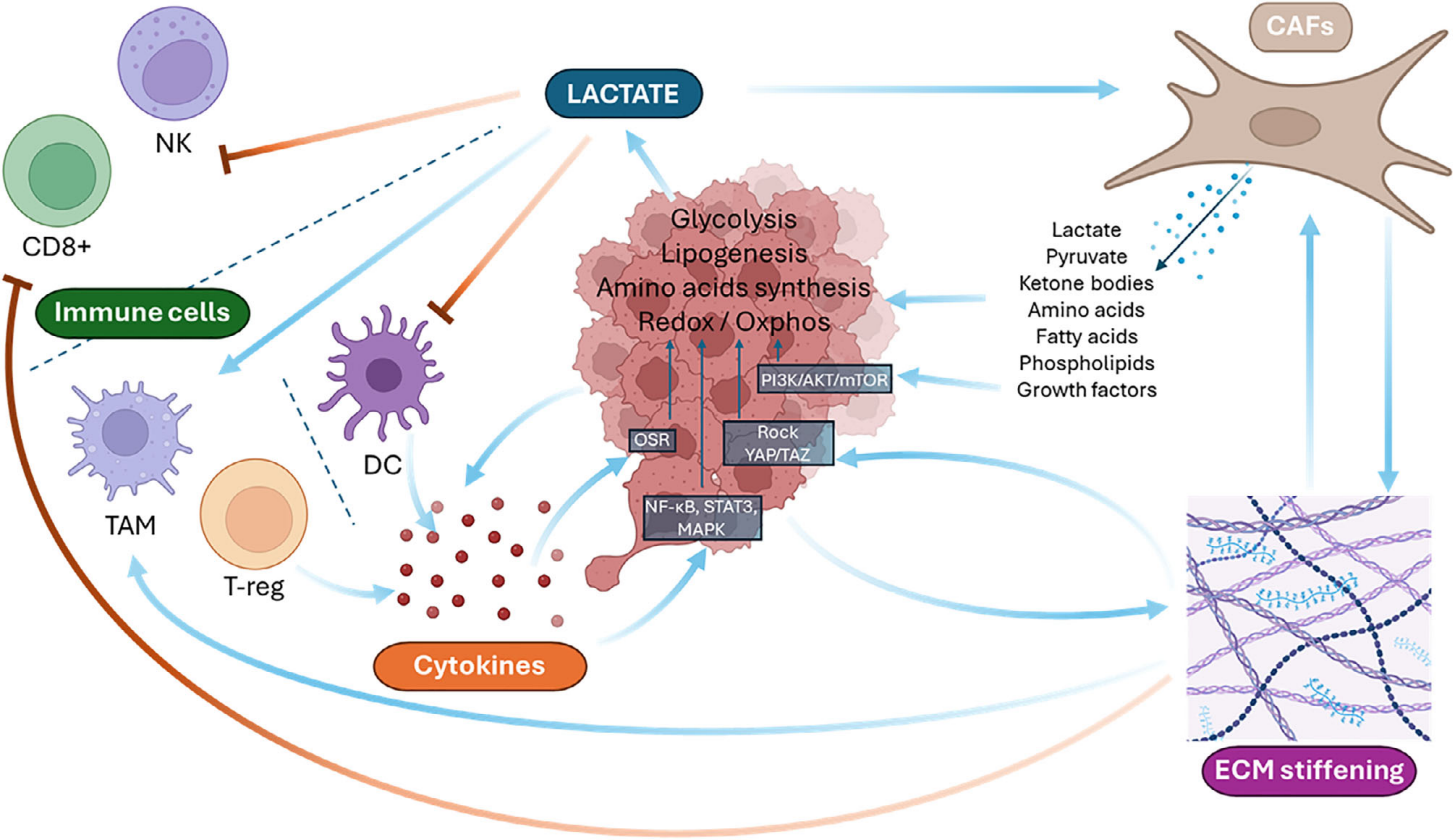
Bidirectional metabolic communications between cancer cells and the tumor microenvironment. Tumor cells actively remodel their surroundings through secretion of metabolites. In turn, stomal cells provide nutrients, growth factors, and inflammatory signals that reinforce tumor metabolic plasticity. These reciprocal interactions create a dynamic ecosystem sensed and controlled by p53. NK = natural killer cells; CAF = cancer‐associated fibroblasts; TAM = tumor‐associated macrophages; T‐Reg = regulatory T‐cell; DC = dendritic cells; OSR = oxidative stress response; ECM = extracellular matrix.

Thus, stromal metabolism should not be viewed simply as a consequence of tumor evolution, but also as a determinant of p53 functional state. Collectively, stromal, immune, vascular, and mechanical components of the TME continuously exchange metabolites, cytokines and biomechanical cues with cancer cells, creating self‐reinforcing adaptive circuits (Figure 2). The integration of these diverse extracellular and intracellular signals requires central regulatory hubs capable of coordinating context‐dependent metabolic responses.

### 3.2 WT p53 as a Stress Microprocessor of the Metabolic/TME Ecosystem

Metabolic and mechanical stresses are relayed to p53 via key sensors pathways. Nutrient‐deprived and hypoxic TME is a hallmark of solid tumors. Nutrients shortage activates AMPK and inhibits mTORC1, activating p53α through phosphorylation and relieving anabolic pressure, while amino acid deprivation triggers the eIF2α‐dependent integrated stress response, thereby promoting cell‐cycle arrest and metabolic adaptation [57]. These mechanisms are further supported by SMAR1‐dependent induction of Δ40p53α upon glucose deprivation, triggering a cell‐cycle arrest and pro‐metabolism transcriptional program [24]. In physiological conditions, this helps to maintain cells and tissue homeostasis in response to short‐term metabolic stress. However, in pathological context, this mechanism increases cancer cells’ resilience. In parallel, hypoxia regulates p53α in a similar context‐dependent manner. Mild hypoxia stabilizes p53α through stress kinase signaling and modulation of MDM2 activity, whereas severe or prolonged hypoxia suppresses p53α function via HIF‐1–dependent mechanisms and altered protein turnover [57]. Hence, crosstalk between p53α and HIF‐1α helps determine whether cells adapt or undergo apoptosis. In solid tumors frequently facing chronic hypoxia, this represents a mechanism to suppress p53 tumor‐suppressive activity. Interestingly, it was recently reported that short‐term nutrient deprivation and hypoxia lead to substantial upregulation of the mRNAs encoding for Δ40p53α, Δ133p53α, and Δ160p53α isoforms in HepG2 tumorspheres [25]. This expression correlates with pro‐survival programs, where apoptosis and cell‐cycle arrest genes are downregulated (i.e., Bax, p21) while survival and proliferation markers, such as BCL2 and PCNA, are upregulated. Furthermore, hypoxia and nutrient shortage were shown to induce stem cell‐like plasticity programs [107], which is a feature associated with Δ133p53α and Δ133p53β [73, 74, 75], and represents a pro‐tumorigenic function of WT p53.

In inflammatory settings, crosstalk between p53 and NF‐κB or STAT3 can be either antagonistic or cooperative, allowing p53 to temper excessive cytokine‐driven survival signaling while preserving redox homeostasis through induction of antioxidant and metabolic target genes [108]. In this crosstalk, p53α is assisted by p53β and Δ133p53α isoforms, which can respectively enhance or inhibit p53α‐mediated senescence and establish an isoform‐dependent control of the senescence‐associated secretory phenotype (SASP), including IL6 and IL11 [67, 68]. This is particularly important as IL6 and IL11 activate STAT3 in stromal CAFs [69], leading them to secrete fatty acids and phospholipids [101]. This represents an ambivalent activity of the WT p53 pathway as senescence induction in some cancer cells slows down tumor progression, but the SASP‐induced stromal changes will sustain proliferation of the non‐senescent cancer cells. In addition, WT p53 promotes the degradation of TREX1, a DNA exonuclease, resulting in cytosolic dsDNA accumulation, which activates the innate immunity cGAS/STING pathway stimulating IFN‐I production [70].

ECM stiffness sensing and modulation is also regulated by WT p53, as the Δ133p53 isoforms shape ECM by regulating the expression and deposition of laminin α3, laminin α5, fibronectin, and Extra Domain A(EDA)‐fibronectin in response to ECM mechanical cues [28]. In this context, loss of Δ133p53 isoforms expression resulted in increased stress fibers formation in 2D and loss of polarized structures in 3D in both non‐malignant and malignant breast cells. Importantly, loss of polarized structures is associated with malignant and invasive breast cancer cells, and this Δ133p53 isoforms‐dependent mechanism, therefore, represents a tumor‐suppressive function [28]. Interestingly, this work also showed that the expression level of the different p53 isoforms is regulated depending on the culture conditions (i.e., 2D vs. 3D and matrix stiffness), suggesting that p53 isoforms expression is also regulated by mechanical cues [28]. Recent work demonstrated that Δ40p53α is induced in cirrhotic liver, and interacts with canonical p53α and Phospho‐SMAD3 to increase collagen III expression and deposition [27].

Thus, in addition to its cell‐autonomous functions, WT p53 shapes the metabolic behavior of stromal and immune compartments. By limiting glycolysis, ROS accumulation and inflammatory cytokine production while promoting antioxidant programs, WT p53 reduces CAF activation, restrains immunosuppressive macrophage polarization and preserves anti‐tumor immune function. Conversely, loss of WT p53‐mediated metabolic homeostasis promotes lactate accumulation, chronic inflammation and extracellular matrix remodeling that progressively reinforce tumor‐supportive stromal phenotypes, impair effector T cell and NK cell function, and immune cells infiltration [11, 102, 103].

### 3.3 Post‐Translational Modification as a Built‐in p53 Tuner

The reciprocal metabolic interactions described above establish the extracellular context within which the p53 pathway operates. Rather than acting solely as a cell‐autonomous metabolic regulator, p53 continuously integrates metabolic, inflammatory, hypoxic and mechanical information generated by stromal and immune compartments before reciprocally reshaping these same compartments through transcriptional and metabolic reprogramming. The p53 pathway is indeed exquisitely sensitive to fluctuations in nutrient availability, redox balance and energy stress. Integration of stress signals impacts p53 stability and activities through post‐translational modifications (PTMs). For instance, all p53 isoforms are ubiquitinated and degraded by the proteasome with different kinetics [109]. Importantly PTMs are tightly coupled to metabolite availability, enabling p53 to function as a metabolic sensor. For example, p53 acetylation is directly dependent on intracellular acetyl‐CoA levels, which fluctuate with glucose, fatty acid and acetate metabolism, and activate p53 tumor suppression through cell‐cycle arrest and apoptosis [110]. Conversely, SIRT1‐mediated deacetylation requires NAD^+^, linking p53 activity to cellular redox and energy state, and inhibiting p53‐dependent apoptosis and radiosensitivity [111]. Methylation of p53 lysines and arginines depends on S‐adenosylmethionine (SAM), the principal methyl donor produced by one‐carbon metabolism, connecting methionine and folate cycle flux and exogenous serine/glycine availability to p53 transcriptional specificity [63, 112]. Methylation of p53 can either enhance or inhibit its tumor‐suppressive function depending on which p53 residue is modified and the modification type (mono‐, bi‐, or tri‐methylation). In addition, O‐GlcNAcylation of p53 by O‐GlcNAc transferase uses UDP‐GlcNAc generated through the hexosamine biosynthetic pathway, integrating glucose, and glutamine availability into p53 regulation [113]. High Ca2+ concentration activates the Peptidylarginine Deiminases (PADIs), including PADI4, which citrullinates at least two arginine residues in mouse p53, influencing its oligomerization and promoter selectivity, reinforcing its tumor suppressive activity [114]. This shows that metabolites’ availability is inherently bound and integrated into p53‐dependent cell fate decisions.

The ability of p53 to function as an adaptive processor, therefore, depends not only on the nature of the extracellular cues it receives, but also on metabolite‐dependent PTMs that fine‐tune its stability, localization, and transcriptional output.

The p53 pathway should, therefore be viewed as a context‐dependent adaptive processor rather than a constitutively tumor‐suppressive pathway. Its biological output is therefore not dictated by a binary molecular switch. Still, it emerges from the integration of TP53 status (WT, mutant, and isoforms expression) and metabolic, inflammatory, mechanical, and genotoxic signals, whose combined intensity and persistence determine whether WT p53 activates homeostatic programs such as cell‐cycle arrest, apoptosis and DNA repair, or instead promotes stress adaptation, tissue remodeling and cellular plasticity. This flexible and context‐dependent nature of the p53 pathway makes it very difficult to confine any single isoform (including canonical p53α) to a strict tumor‐suppressor or tumor‐promoting role (Table 2). Overall, WT p53 engages adaptive programs that preserve cell viability and tissue integrity under transient stress even though some specific functions may inadvertently promote cancer cell persistence, plasticity or therapy resistance when these same mechanisms are chronically engaged during tumor evolution (Table 1 and 2).

**TABLE 2:** Metabolic and microenvironmental functions of the major p53 isoforms during tumor evolution.

p53 isoform	Major inputs sensed	Major metabolic functions	Effects on the TME	Tumor suppressive / oncogenic role	Ref.
p53α (canonical)	Glucose, amino acids, fatty acids, acetate, acetyl‐CoA, NAD+, SAM, UDP‐GlcNAc, Ca2+, ROS, hypoxia, matrix stiffnessB2	Suppresses glycolysis; promotes mitochondrial respiration, oxidative phosphorylation, fatty acid oxidation and redox homeostasis; inhibits PI3K/AKT/mTOR; integrates AMPK, mTOR and HIF signaling; metabolic sensor through PTMs (e.g. acetylation, methylation, O‐GlcNAcylation)	Restrains inflammatory signaling; activates cGAS/STING through TREX1 degradation; regulates ECM remodeling; limitsTME‐activating metabolites secretion (e.g. lactate); Enhances SASP; promotes CAF activation through IL‐6/IL‐11 secretion	**Predominantly tumor suppressive**, although context‐dependent SASP secretion may indirectly support tumor progression and may favor survival under chronic metabolic stress by increasing metabolic adaptation	[22, 23, 24, 36, 56, 57, 63, 65, 67, 68, 69, 70, 71, 110, 111, 112, 113, 114, 115]
Δ40p53α	Glucose deprivation, nutrient stress, hypoxia, matrix stiffness	Coordinates metabolic adaptation during glucose deprivation; cooperates with p53α to induce TIGAR, p21 and 14‐3‐3σ; promotes mitochondrial DNA maintenance via POLγ; reduce apoptosis and cell cycle arrest while promoting survival and proliferation programs	Promotes collagen III deposition in fibrotic liver through SMAD3	**Context‐dependent**; tumor suppressive during acute stress, but may favor survival under chronic metabolic stress by increasing metabolic adaptation	[17, 25, 26, 27]
Δ133p53α	Mechanical cues, inflammatory signaling, nutrient stress, hypoxia	Promotes metabolic adaptation under nutrient stress; activates RhoA/ROCK signaling; reduces p53‐induced senescence and associated inflammatory programs (SASP)	Shapes ECM composition; regulates laminin and fibronectin deposition; maintains structure polarization; reduces CAF activaton through reduced IL‐6/IL‐11 secretion (SASP)	**Context‐dependent**; helps to maintain normal tissue homeostasis by promoting cell cycle arrest and DNA repair over senescence and apoptosis, non‐transforming and maintains structure polarization, but prevents senescence and promote stemness of cancer cells	[17, 19, 25, 28, 30, 67, 68, 69, 73, 75, 79]
p53β	Senescence‐inducing stress, e.g. DNA damage, oncogene activation, oxydative stress	Limited evidence for direct metabolic regulation; synergizes with p53α to induce p21 and NFκB	Enhances senescence and SASP; promotes CAF remodeling through IL‐6/IL‐11 secretion (SASP)	**Mainly tumor suppressive** through senescence, but context‐dependent pro‐tumorigenic through SASP	[17, 67, 68, 69]
Δ133p53β	N/A	Associated with metabolic programs supporting migration and stem‐like plasticity; promotes amoeboid motility	Facilitates invasion through blood‐brain barrier; associated with metastatic dissemination; contributes to inflammatory microenvironment	**Tumor promoting**	[17, 19, 60, 61, 74, 79]
Missense mutant p53α	Nutrient deprivation, hypoxia, ROS, mevalonate pathway, acetyl‐CoA, NAD+, SAM	Enhances glycolysis, glutamine utilization, lipid synthesis, mevalonate pathway, antioxidant responses and mitochondrial adaptation; regulates PDK1, LAT1, serine synthesis and one‐carbon metabolism; PTMs remain metabolically regulated	Promotes CAF activation, ECM remodeling, YAP/TAZ activation, inflammatory cytokine production, immune suppression, metastatic niche formation	**Tumor promoting**	[6, 7, 15, 29, 31, 32, 33, 34, 35, 58, 59, 64, 66, 72, 116, 117, 118, 119, 120, 121, 122, 123]
Missense mutant Δ133p53α R273H	N/A	Enhances proliferation and invasion; reduces p53‐induced senescence and associated inflammatory programs (SASP)	Suppresses anti‐tumor immunity through IDO1/kynurenine	**Tumor promoting**	[30]
STOP‐lost β mutant	N/A	Strongly binds to p53α (WT) to increases p21 and decrease Bax and IGFBP3 expression	N/A	**Tumor promoting—**early onset tumor development as germline mutation	[83]

Importantly, this regulatory layer operates in both WT and mutant p53 contexts. While WT p53 operates as a finely tuned metabolic and microenvironmental stress microprocessor, tumor‐associated mutations rewire this system, converting it into a driver of adaptive and pro‐tumorigenic responses.

### 3.4 Mutant p53 as a Rewired Mechano‐Metabolic Microprocessor

In this context, mutant p53 functions as a rewired microprocessor that integrates mechano‐metabolic cues, while redirecting cellular responses toward tumor‐promoting adaptation. Similarly to WT p53, the activity of mutant p53 is regulated by PTMs, which are tightly linked to metabolite availability as described above. For instance, acetylation of mutant p53 by p300/CBP enhances its transcriptional activity and oncogenic potential, while mutant p53 can in turn promote p300 autoacetylation, reinforcing aberrant gene expression programs [116, 117]. In parallel, mutant p53 stability is tightly controlled by ubiquitination and proteasomal degradation pathways, which necessitate prior deacetylation by NAD^+^‐dependent sirtuins, as well as by interactions with molecular chaperones such as HSP90, which protect it from degradation and sustain its accumulation in tumors [31, 117]. SAM‐dependent methylation of mutant p53 modulates its transcriptional output by altering its interaction with chromatin and transcriptional cofactors, thereby influencing promoter selectivity and gene expression programs [117, 118].

Furthermore, cues from the TME (e.g., hypoxia, inflammation) also regulate mutant p53 expression and activity. In breast cancer, amino acid shortage promotes mutant p53 stabilization, which induces the expression of L‐type amino acid transporter 1 (LAT1)/CD98 heavy chain heterodimer and serine‐synthesis‐pathway enzymes [64]. By increasing essential amino acids intake and de novo serine/glycine synthesis in response to deprivation, mutant p53 senses and adapts the cancer cell metabolism to nutrient deprivation [64]. Interestingly, this offers therapeutic opportunities by targeting the mechanisms responsible for this adaptive mechanism. Recent evidence also shows that missense mutant p53 and pyruvate dehydrogenase kinase 1 (PDK1) increase each other expression/activity and thus promote tumor progression in breast cancer [58]. PDK1 is a major glycolysis regulator, particularly under low nutrient or hypoxic conditions, and its stimulation allows mutant p53 cells to adapt and develop in these conditions [72].

Mutant p53 also influences these mechanisms as it cooperates with inflammatory transcription factors to amplify NF‐κB‐driven cytokine expression, sustaining chronic inflammation, and cancer cell metabolic adaptation [29]. By reprogramming metabolic and inflammatory circuits, mutant p53 also influences myeloid cell recruitment and T cell dysfunction, by altering antigen presentation and immune cell crosstalk [7], to deepen immune suppression during tumor progression. This is further supported by mutant Δ133p53α direct upregulation of IDO1 expression [30], known for promoting tryptophan catabolism and kynurenine pathway activation, and leading to cytotoxic T cell activity suppression and immune escape [3].

Mechanical cues also feed into mutant p53 regulation as ECM stiffness and integrin‐mediated cytoskeletal tension influence p53 stability through mechanotransduction pathways [11]. In particular, the mevalonate pathway activates RhoA/actin‐dependent transduction, which leads to the accumulation of mutant p53 by protecting it from ubiquitin‐mediated proteolysis [35]. This protection depends on RhoA geranylgeranylation, a PTM which may offer new therapeutic opportunities by using statins or geranylgeranyl transferase inhibitors [35].

The mutant p53/mevalonate/RhoA axis also triggers YAP/TAZ signaling, consequently increasing cancer cells’ stemness and proliferation [34, 35]. In return, mutant p53 is highly potent in regulating ECM changes, particularly through direct induction of metalloproteinases and the repression of their tissue inhibitor, TIMP3 [66]. Furthermore, mutant p53 interacts with HIF‐1α and induces the expression of miR‐30d, thereby promoting the release of a malignant secretome that remodels the ECM and activates cancer‐associated fibroblasts (CAFs), enhances mechanotransduction signaling, and ultimately contributes to metastatic dissemination [6].

This demonstrates that, far from abolishing its metabolic and microenvironmental stress‐processing functions, p53 mutations rewire the protein into a mechano‐metabolic microprocessor that facilitates cancer cell adaptation and survival (Table 1). Mutant p53 acts not only as an intracellular metabolic regulator, but also as an organizer of metabolic communication within the TME. Through increased secretion of cytokines, extracellular vesicles, and metabolites, mutant p53 reprograms CAFs, TAMs, endothelial cells and adaptive immune cells toward tumor‐supportive metabolic states, establishing feed‐forward circuits that amplify metabolic plasticity during tumor progression. Beyond its direct effects on metabolic and signaling pathways, mutant p53‐driven reprogramming converges on epigenetic regulation of cellular plasticity, linking metabolic flux to chromatin state.

### 3.5 Metabolic‐Epigenetic Control of Cancer Cell Plasticity and Transposable Element Regulation by p53

As a key factor in tumor evolution, cancer cell plasticity is increasingly recognized as a dynamic process driven by the interplay between metabolic rewiring and epigenetic regulation in response to microenvironmental cues, such as hypoxia, nutrient limitation, and inflammatory signals. Different intermediates of metabolic pathways donate chemical groups used to modify cellular molecules, including DNA, RNA, and proteins, thereby regulating different cellular processes (e.g. signaling, chromatin architecture, transcript stability, and organelle function) impacting cell adaptation to microenvironmental cues [115, 119, 120, 124, 125, 126]. Prominent examples include: Acetyl‐CoA fueling histone acetylation; one‐carbon metabolism regulating the availability of SAM and thus methylation of DNA, RNA, histones, and non‐histone proteins; TCA intermediates (e.g. α‐ketoglutarate, succinate, fumarate) coupled with the activity DNA/histone demethylases [127]. In cancer, different oncogenes (e.g. RAS, mTOR, MYC, YAP) can rewire these metabolic pathways, increasing glucose, glutamine, lipid, and amino‐acid fluxes to support acetyl‐CoA, NADPH, nucleotide, and one‐carbon metabolism [11]. Also, mutant p53 promotes serine‐glycine synthesis and amino‐acid uptake, linking nutrient stress to SAM‐dependent methylation [64]. These oncogene‐metabolism circuits epigenetically reshape the transcriptome and proteome, thereby promoting cancer cell adaptation, inhibiting the production of potentially immunogenic nucleic acids and peptides, suppressing immunity pathways, and remodeling the TME. Through these pathways, oncogene‐driven metabolic rewiring also influences tumor response to treatments and represents a potentially targetable vulnerability.

In the cell adaptation to microenvironmental cues, a key role is played by the epigenetic regulation not only of protein‐coding genes, but also non‐ coding genes sequences, including repetitive sequences, such as transposable elements (TEs). TEs account for nearly half of the human genome, and are key regulators of cancer cell fitness, plasticity and immunogenicity [126, 128]. They include DNA and retroTEs, some of which can mobilize in the genome, harbor regulatory sequences, and can generate non‐canonical chimeric transcripts. Also, TE species in the cytoplasm can potently activate innate immunity (e.g. cGAS/STING/IFN‐I) [129, 130]. Due to these features, TEs can cause DNA damage/mutations, reshape the transcriptome/proteome and trigger immune reactions; thus, in normal cells, they are repressed (post)transcriptionally, crucially by DNA/histone methylation [128, 131]. In cancer cells, TEs are frequently reactivated and can become pro‐oncogenic (e.g. by causing genome instability and oncogene activation/tumor suppressor inactivation) [132]. However, cancer cells can cope with TE immunogenicity by oncogene‐dependent TE epigenetic repression and inhibition of immune pathways [133].

In this context, p53 emerges as a central integrator of metabolic and epigenetic signals, and its mutation profoundly alters cancer cell plasticity and immunogenicity. When WT p53 is activated in response to metabolic or genotoxic stress, it functions as a tumor‐suppressive rheostat that restrains anabolic growth and preserves chromatin fidelity. WT p53 regulates multiple metabolic pathways whose intermediates provide chemical groups for regulatory modifications, including glycolysis, mitochondrial respiration, and one‐carbon metabolism, thereby affecting acetyl‐CoA‐dependent acetylation, NAD^+^‐dependent deacetylation, and SAM‐dependent methylation of nucleic acids and proteins. In cancer cells, mutant p53 not only rewires metabolic pathways and thus reshapes epigenetic and transcriptional programs through interactions with chromatin modifiers and transcription factors, promoting aberrant expression not only of coding genes, but also of non‐coding RNAs and TEs, such as LINE‐1 retrotransposons [119, 120, 121, 122].

Interestingly, the regulation of one‐carbon metabolism by p53, including control of SAM availability, has been recently shown to link metabolic flux to epigenetic modifications, such as DNA methylation, which control the expression of coding and TE transcripts [115]. WT p53 sustains one‐carbon metabolism and methionine‐cycle capacity to maintain adequate SAM, thereby supporting DNA and histone methylation at constitutive heterochromatin and restraining unscheduled transcription of repetitive elements, thus maintaining genome integrity [115]. Conversely, p53 mutation reduces SAM, and this weakens heterochromatin methylation, derepresses repetitive elements, and promotes R‐loop‐associated replication stress, thus promoting genome instability, which contributes to cancer cell plasticity [115]. However, mutant p53 has been shown to drive cell adaptation to TE derepression to cope with their potential immunogenicity. For instance, p53‐deficient cells adapt to LINE‐1 expression by upregulating the Fanconi anaemia pathway [120]. Likewise, in premalignant ovarian lesions, where *TP53* mutations have nearly 100% penetrance as cancer‐initiating event [134], p53 loss induces tolerance to chronic viral mimicry and promotes immune evasion by decreasing p300/CBP‐dependent H3K27 acetylation (H3K27ac) at the promoters of interferon‐stimulated genes (ISGs), resulting in reduced ISG expression and diminished secretion of pro‐inflammatory cytokines [71].

Furthermore, in mutant p53 pancreatic ductal adenocarcinoma, LINE‐1‐encoded ORF1p protein has been shown to bind Alu repeat elements RNAs and reduces their expression, thus dampening Alu‐derived dsRNAs activation of viral mimicry IFN‐I [123]. In addition, mutant p53 has been shown to suppress innate immune signaling by inhibiting TBK1 and the consequent activation of the cGAS‐STING‐IFN‐I innate immunity pathway [33].

Thus, oncogene‐dependent rewiring of the metabolism‐epigenetic landscape plays a crucial role in tumor evolution by shaping cancer cell plasticity and immunogenicity, and in this context, mutant p53 is a prominent example of oncogene‐dependent balance of pro‐ and anti‐tumorigenic consequences of TE activity in cancer cells. This represents a vulnerability that can be exploited to stimulate anti‐tumor immune responses, as suggested by the detection of circulating antibodies against LINE‐1‐encoded antigens in cancer patients [135].

Together, all these elements highlight a dynamic metabolic–microenvironmental interplay which generates a self‐reinforcing niche that supports invasion, immune evasion, and metastatic dissemination. Through its dual capacity to sense stromal and intracellular perturbations and to reciprocally remodel both tumor cells and their microenvironment, the p53 pathway stands at the crossroad of this interplay as a mechano‐metabolic‐sensor and regulator (Table 2).

## 4 Metastasis and Metabolic Dependencies

Cancer cell metabolic state is linked to the tissue of origin, and its rewiring is essential for primary tumors to adapt and survive [136]. The development of metastasis in a different tissue requires an even greater degree of adaptability to survive invasion, circulation, seeding, and colonization of distant organs. Importantly, recent evidence shows that metastases maintain lineage‐specific metabolic programs rather than fully adopting those of the host tissue, providing an explanation to why most primary tumor types metastasize preferentially to a subset of target tissues [136, 137].

### 4.1 Metabolic Control of Invasion and Early Metastatic Dissemination

In primary tumors, increased actomyosin contractility and integrin signaling due to ECM stiffening have been shown to enhance mitochondrial activity and ROS production, thereby promoting invasive behavior [76, 77]. This mechanical‐metabolic coupling supports epithelial‐mesenchymal transition (EMT), matrix degradation and motility, all of which are energetically demanding processes requiring flexible substrate utilization [77]. These adaptations are critically influenced by oncogenic pathways, including mutant p53, which coordinate cytoskeletal remodeling, metabolic rewiring and transcriptional programs that collectively promote invasive phenotypes and metastatic competence [29]. For instance, mut‐p53/HIF1α/miR‐30d‐driven Golgi tubulo‐vesiculation promotes a pro‐malignant secretome that contributes to the establishment of a permissive microenvironment for metastatic dissemination [6].

Metabolic plasticity becomes even more critical during dissemination, where circulating tumor cells must withstand oxidative stress and nutrient deprivation in the bloodstream. Accordingly, redox buffering systems, including glutathione metabolism and NADPH‐generating pathways, are essential for metastatic competency, as excessive ROS accumulation limits survival during hematogenous spread [78]. In this context, WT p53 can restrain invasion by limiting glycolysis, lipogenesis, and oxidative stress, thereby imposing metabolic checkpoints that suppress metastatic progression. Nevertheless, metastatic traits can still emerge in tumors expressing WT p53. Notably, WT Δ133p53 isoforms were shown to activate the RhoA‐ROCK pathway, promoting actin‐myosin cytoskeleton remodeling and stress fibers formation, thus promoting migratory and invasive phenotypes in colorectal cancer cells [79]. Furthermore, in breast tumors increased expression of the WT Δ133p53β isoform has been shown to correlate with invasion and metastatic dissemination to the brain [60]. Mechanistically, Δ133p53β promotes the loss of adhesive structures and acquisition of a rounded amoeboid morphology [61], while facilitating invasion across the blood–brain barrier [60]. Collectively, these findings highlight that, even in WT p53 contexts, the unbalancing of isoforms expression drives pro‐metastatic transcriptional and metabolic programs to enable adaptation to dissemination and colonization of distant niches.

Following dissemination, tumor cells must transition from survival in circulation to successful adaptation and sustained growth within distant organ microenvironments, a process that requires a second wave of metabolic reprogramming.

### 4.2 Metabolic Control of Metastatic Colonization and Organ‐Specific Adaptation

In metastatic colonization, disseminated tumor cells must adapt to the unique metabolic, structural, and immunological constraints of the distant organ microenvironment to establish sustained proliferative growth [138]. In this context, mutant p53 is particularly efficient in allowing colonizing cells to adapt to these tissue‐specific limitations. For instance, in organs such as the lung and brain, where oxygen tension and nutrient availability fluctuate, the ability to maintain redox homeostasis becomes particularly important for metastatic growth and therapeutic resistance. Antioxidant pathways activated downstream of mutant p53α help tumor cells tolerate oxidative stress and sustain mitochondrial function during metastatic colonization [29, 59, 139]. These adaptations not only promote survival, but may also contribute to resistance against therapies that rely on oxidative stress–induced cytotoxicity. In parallel, mutant p53 has been shown to promote metabolic rewiring and tumor cell migration through regulation of metabolic enzymes, such as PDK1 [58]. By modulating metabolism, cytokine signaling and redox regulation, mutant p53α establishes a microenvironment that favors metastatic expansion, while suppressing anti‐tumor immune responses [6, 29, 58, 59]. Recent genomic and metabolic analyses further support that alterations in the p53 pathway facilitate the adaptation of metastatic cells to a restrictive microenvironment. In breast cancer models, loss of p53 function promotes upregulation of fatty‐acid synthesis, allowing metastatic cells to generate lipids required for membrane synthesis and energy production in the lipid‐poor brain microenvironment [62]. These metabolic adaptations enhance the ability of disseminated tumor cells to proliferate within the brain and establish macro metastases, illustrating how p53‐dependent metabolic regulation influences organ‐specific metastatic colonization [62]. This is further supported by recent report that increased fatty acids synthesis stabilizes MYC through acetylation which, in turn, activates proline synthesis and collagen production, hence favoring colorectal cancer metastasis to the liver [140].

Although metabolic adaptation enables successful metastatic colonization, not all disseminated tumor cells immediately sustain proliferative growth. In many cases, cells enter a reversible state of dormancy, in which survival is maintained under persistent metabolic and microenvironmental constraints.

### 4.3 Metabolic and Mechanical Regulation of Metastatic Dormancy

In metastatic dormancy, disseminated tumor cells persist in a reversible non‐proliferative state characterized by metabolic quiescence and enhanced stress tolerance under sustained microenvironmental constraints, allowing long‐term survival before metastatic relapse. Dormancy is regulated by both metabolic and mechanical cues within the metastatic niche. Metabolically, dormant cells often adopt a low‐proliferative state characterized by reduced anabolic activity and increased stress‐adaptation pathways, including autophagy and oxidative stress management [141]. This is important as excessive ROS accumulation can trigger apoptosis, while insufficient ROS signaling may impair proliferative reactivation [78]. Also, soft or growth‐restrictive microenvironments can maintain quiescence, whereas stiffening and inflammatory remodeling promote metabolic reactivation and metastatic outgrowth [141]. In this context, p53 functions as a metabolic checkpoint that can either enforce dormancy through cell‐cycle arrest and oxidative control or, when mutated, permit metabolic escalation, and proliferative re‐entry [139, 142].

**FIGURE 3:**
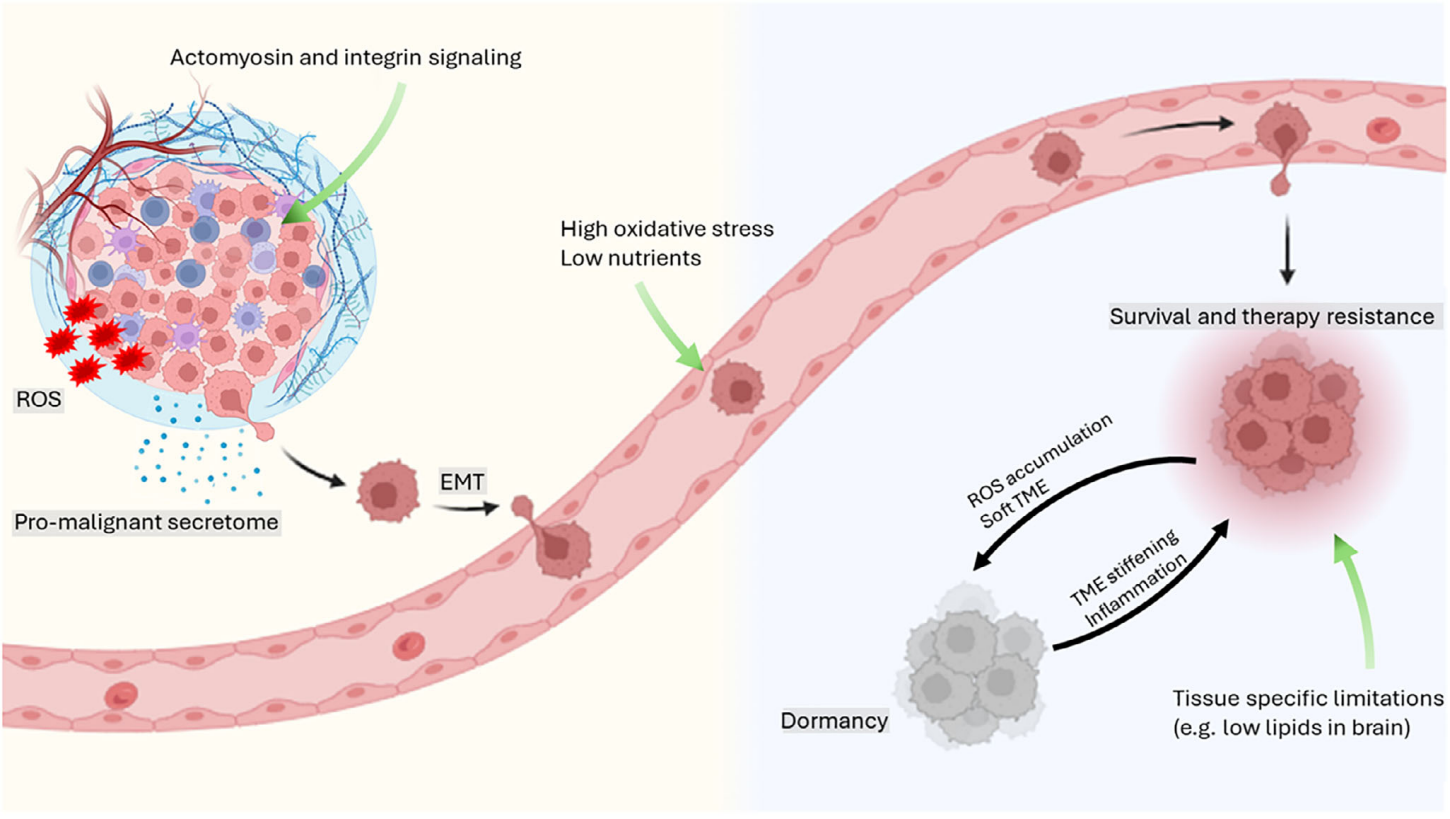
Metabolic dependencies across the metastatic cascade. Metastasis requires stage‐specific metabolic adaptations. At initiation, during circulation, and colonization requires high plasticity to survive dissemination, adapt to and new tissue environment, and dormancy. p53 contributes to these transitions by regulating redox homeostasis, metabolic checkpoints, and dormancy programs. Loss or mutation of p53 may facilitate metastatic reactivation by promoting metabolic plasticity. ROS = reactive oxigen species; EMT = epithelial to mesenchymal transition.

Hence, metabolic plasticity and mechanical signals underlie each step of the metastatic cascade (Figure 3). By coordinating lipid metabolism, redox homeostasis, and interactions with inflammatory and mechanical cues, the p53 pathway emerges as a rheostat whose functional state (WT, mutant, isoforms balance) critically influences invasion, organ‐specific colonization, dormancy and therapeutic resistance.

## 5 Conclusions and Prospects

The metabolic plasticity that enables tumor progression also exposes therapeutic vulnerabilities. Many tumors become dependent on specific metabolic pathways (e.g., lipid synthesis, amino‐acid metabolism, or redox regulation) to survive under microenvironmental stress. These liabilities can be targeted pharmacologically or exploited to sensitize tumors to chemotherapy, radiotherapy and immunotherapy [143]. In this context, targeting the p53 pathway, or the metabolic dependencies sustained by p53 dysfunction, may provide new therapeutic opportunities [139].

A particularly promising area is the therapeutic exploitation of (mutant) p53‐rewired metabolic regulators rather than mutant p53 itself. For instance, GOF mutant p53 has been linked to activation of the mevalonate pathway, supporting tumor growth, metastatic behavior, and therapy resistance; this provides a rationale for repurposing statins or targeting downstream mevalonate pathway components [144, 145]. Mechanistically, statins also destabilize GOF mutant p53 by promoting both CHIP‐ and MDM2‐mediated degradation through disruption of the mutant p53‐DNAJA1 and mutant p53‐Hsp90 interactions, respectively, connecting metabolic pathway inhibition with mutant p53 proteostasis. Another relevant axis is mutant p53‐mediated suppression of AMPK, a metabolic checkpoint that normally constrains anabolic metabolism; GOF mutant p53 can inhibit AMPK activation under energy stress, thereby sustaining glycolytic and invasive phenotypes [146]. These observations suggest that AMPK activation, mTOR/anabolic pathway inhibition, or energetic stress sensitization may be rational strategies in mutant p53‐expressing tumors, although clinical studies have not yet robustly validated this approach in TP53‐mutant‐selected cohorts.

A second targetable layer involves lactate metabolism and tumor‐immune metabolic competition. Loss of p53 function promotes MCT1 expression and lactate export under hypoxia, while MCT1 expression is elevated in p53‐mutant, hypoxic breast tumors and is associated with poorer clinical outcomes [65]. This provides a rationale for targeting lactate transport in p53‐defective tumors, particularly because lactate accumulation contributes to extracellular acidification and immune suppression. The MCT1 inhibitor AZD3965 has completed first‐in‐human Phase I testing in advanced cancer; the trial evaluated safety, pharmacokinetics and recommended dosing, and the published dose‐escalation study reported target engagement and an RP2D of 10 mg twice daily [147]. Similarly, LDH inhibition has been proposed as an immunometabolic strategy: A 2024 preclinical study showed that LDH inhibition reduced tumor‐cell glucose uptake, while increasing glucose uptake and proliferation of tumor‐infiltrating T cells and improved the activity of immune‐checkpoint blockade in murine melanoma and colon cancer models [148].

Glutamine metabolism and ferroptosis represent additional, mechanistically connected vulnerabilities. Glutaminase enzymes support glutamine conversion to glutamate, fueling biosynthesis and antioxidant capacity; GLS is generally associated with tumor growth, whereas GLS2 can behave as either a tumor suppressor or tumor promoter depending on cancer context [149]. The BeGIN Phase II trial of telaglenastat/CB‐839 (ClinicalTrials.gov ID NCT03872427) provides an example of genotype‐guided glutaminase inhibition, although the trial selects NF1, KEAP1/NRF2, and STK11/LKB1 alterations rather than TP53 mutations. Ferroptosis is also relevant because p53 can modulate SLC7A11, SAT1, GLS2, p21, and DPP4‐dependent ferroptosis programs, while GPX4 is a central suppressor of lipid‐peroxidation‐driven cell death [150]. However, ferroptosis‐targeted therapy remains largely preclinical, and the field still faces issues of drug delivery, toxicity, and resistance.

Thus, mutant p53‐rewired metabolism provides multiple therapeutic entry points, but the strongest clinical message is that these targets should be advanced through biomarker‐selected combination strategies rather than treated as universally actionable (mutant) p53 dependencies. Indeed, (mutant) p53 accumulation is highly context dependent and reflects the integration of metabolic and microenvironmental stresses rather than TP53 genotype alone. Therefore, combining genomic information with mutant p53 protein abundance may better identify tumors that are functionally dependent on mutant p53 signaling. Furthermore, beyond genomic profiling, functional biomarkers reflecting p53 pathway activity, including mutant p53 stabilization, p53 isoform balance and metabolic signatures, may prove more informative than TP53 mutation status alone for predicting therapeutic response [17, 19].

**FIGURE 4:**
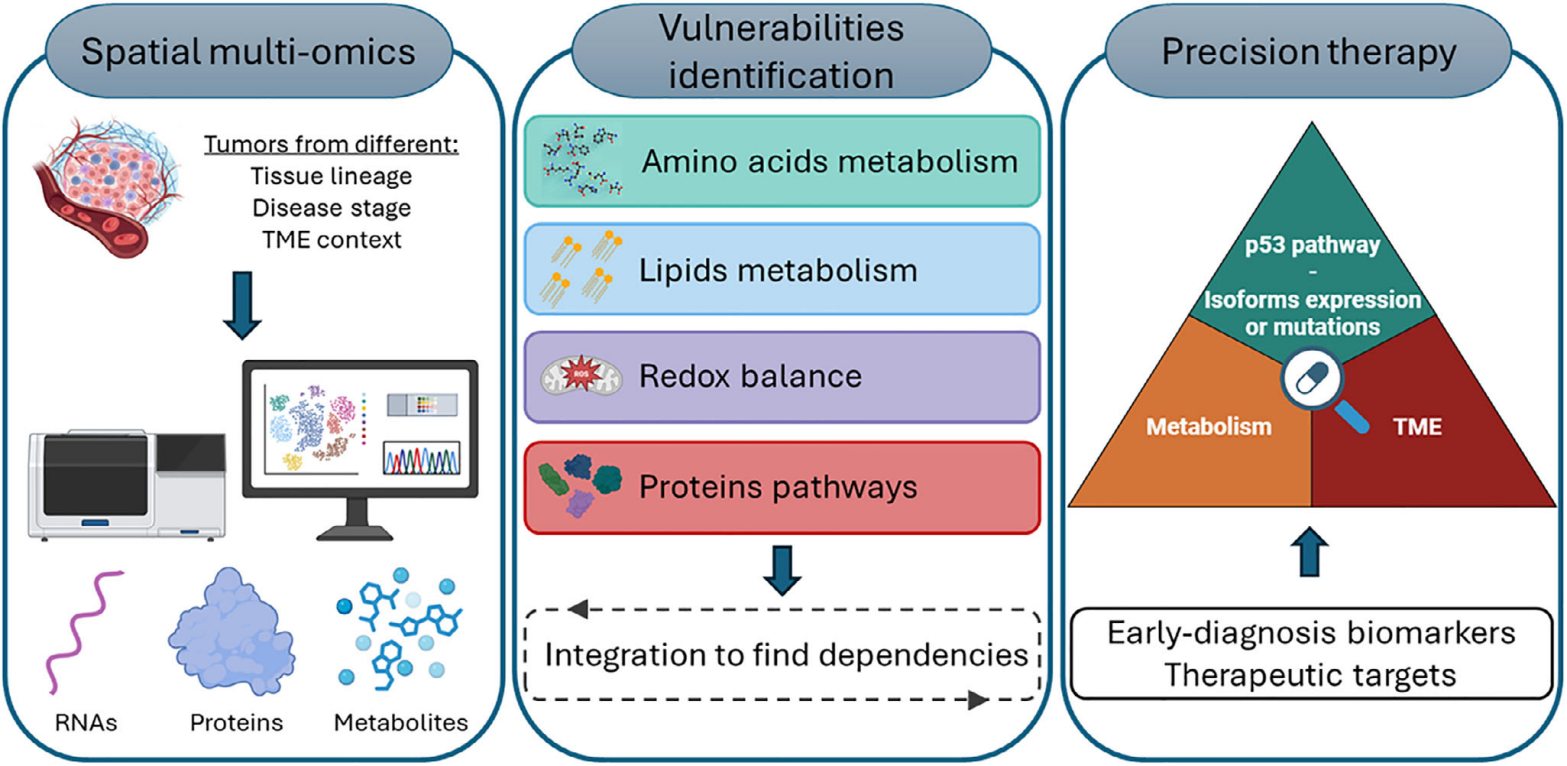
Mapping metabolic ecosystems to identify therapeutic vulnerabilities. Emerging spatial multi‐omics technologies enable simultaneous analysis of transcriptional, proteomic, and metabolic states within intact tumor ecosystems. These approaches may identify metabolic dependencies that can be therapeutically targeted. Integration of p53 status, metabolic context, and microenvironmental information may enable more precise therapeutic strategies aimed at disrupting adaptive tumor ecosystems.

This underscores the need for systematic studies integrating TP53 mutation type (loss‐of‐function versus gain‐of‐function mutations), p53 isoform expression, p53 protein stabilization, tumor metabolic phenotype, and TME composition to establish biologically meaningful patient stratification, such multidimensional profiling will likely identify the subsets of patients most likely to benefit from therapies targeting p53‐dependent metabolic vulnerabilities, rather than considering TP53‐mutant cancers as a homogeneous clinical entity. Recent advances in multi‐omics (transcriptomics, proteomics, and metabolomics) at single‐cell and spatial resolution may provide a big leap forward to dissect this complexity [151]. Spatially resolved studies can chart the dynamic interplay between TME and tumor cell plasticity, which helps to understand heterogeneity and offer new therapeutic perspectives [151]. Importantly, applied to premalignant tissues, these multi‐omics techniques may enlighten the evolution from precancerous states to malignancy and allow the development of early‐diagnosis biomarkers and targeted pharmacological preventive strategies [152]. Integrating spatial multi‐omics across multiple tissue lineages and disease stages will therefore be essential to identify context‐specific vulnerabilities and guide the development of precision therapies targeting the evolving metabolic ecosystems that sustain cancer initiation, growth, and metastasis (Figure 4).

Rather than representing a static tumor suppressor or oncogene, the p53 pathway should increasingly be viewed as an adaptive processor whose activity is dynamically shaped by metabolic and microenvironmental signals. Understanding this context dependence will be essential for translating p53 biology into effective precision oncology.

## Author Contributions

**SMJ and FN**: conceptualization, writing, reviewing, and editing. **RB and GDS**: conceptualization, reviewing, and editing. All authors approved the final article.

## Conflicts of Interest

The authors declare no conflicts of interest.

## Data Availability

Data sharing is not applicable to this article as no datasets were generated or analyzed during the current study.

## References

[1] D. Hanahan , “Hallmarks of Cancer—Then and Now, and Beyond,” Cell 189, no. 8 (2026): 2254–2277, 10.1016/j.cell.2025.12.049. 41616779

[2] K. E. de Visser and J. A. Joyce , “The Evolving Tumor Microenvironment: From Cancer Initiation to Metastatic Outgrowth,” Cancer Cell 41, no. 3 (2023): 374–403, 10.1016/j.ccell.2023.02.016. 36917948

[3] J. A. Wik , E. R. Berge , K. Stromsnes , and B. S. Skålhegg , “Metabolism in the Tumor Microenvironment: Implications for Pathogenesis and Therapeutics,” Frontiers in Immunology 16 (2025): 1610255, 10.3389/fimmu.2025.1610255. 41235226 PMC12605492

[4] J. Son , C. A. Lyssiotis , H. Ying , et al., “Glutamine Supports Pancreatic Cancer Growth Through a KRAS‐Regulated Metabolic Pathway,” Nature 496, no. 7443 (2013): 101–105, 10.1038/nature12040. 23535601 PMC3656466

[5] G. Hoxhaj and B. D. Manning , “The PI3K–AKT Network at the Interface of Oncogenic Signalling and Cancer Metabolism,” Nature Reviews Cancer 20, no. 2 (2020): 74–88, 10.1038/s41568-019-0216-7. 31686003 PMC7314312

[6] V. Capaci , L. Bascetta , M. Fantuz , et al., “Mutant p53 Induces Golgi Tubulo‐Vesiculation Driving a Prometastatic Secretome,” Nature Communications 11, no. 1 (2020): 3945, 10.1038/s41467-020-17596-5. PMC7414119 32770028

[7] D. B. Mahat , H. Kumra , S. A. Castro , et al., “Mutant p53 Exploits Enhancers to Elevate Immunosuppressive Chemokine Expression and Impair Immune Checkpoint Inhibitors in Pancreatic Cancer,” Immunity 58, no. 7 (2025): 1688–1705.e9, 10.1016/j.immuni.2025.06.005. 40592342 PMC12233199

[8] E. J. Kay and S. Zanivan , “The Tumor Microenvironment Is an Ecosystem Sustained by Metabolic Interactions,” Cell Reports 44, no. 3 (2025): 115432, 10.1016/j.celrep.2025.115432. 40088447

[9] P. Altea‐Manzano , A. Decker‐Farrell , T. Janowitz , and A. Erez , “Metabolic Interplays Between the Tumour and the Host Shape the Tumour Macroenvironment,” Nature Reviews Cancer 25, no. 4 (2025): 274–292, 10.1038/s41568-024-00786-4. 39833533 PMC12140436

[10] R. Nakahara , K. Maeda , S. Aki , and T. Osawa , “Metabolic Adaptations of Cancer in Extreme Tumor Microenvironments,” Cancer Science 114, no. 4 (2023): 1200–1207, 10.1111/cas.15722. 36630222 PMC10067430

[11] R. Bertolio , F. Napoletano , and G. Del Sal , “Dynamic Links Between Mechanical Forces and Metabolism Shape the Tumor Milieu,” Current Opinion in Cell Biology 84 (2023): 102218, 10.1016/j.ceb.2023.102218. 37597464

[12] E. M. Pålsson‐McDermott and L. A. J. O'Neill , “Targeting Immunometabolism as an Anti‐Inflammatory Strategy,” Cell Research 30, no. 4 (2020): 300–314, 10.1038/s41422-020-0291-z. 32132672 PMC7118080

[13] M. Demicco , X.‐Z. Liu , K. Leithner , and S.‐M. Fendt , “Metabolic Heterogeneity in Cancer,” Nature Metabolism 6, no. 1 (2024): 18–38, 10.1038/s42255-023-00963-z. 38267631

[14] A. Herms , B. Colom , G. Piedrafita , et al., “Organismal Metabolism Regulates the Expansion of Oncogenic PIK3CA Mutant Clones in Normal Esophagus,” Nature Genetics 56, no. 10 (2024): 2144–2157, 10.1038/s41588-024-01891-8. 39169259 PMC11525199

[15] D. Dibra , M. Gagea , Y. Qi , G. P. Chau , X. Su , and G. Lozano , “p53R245W Mutation Fuels Cancer Initiation and Metastases in NASH‐driven Liver Tumorigenesis,” Cancer Research Communications 3, no. 12 (2023): 2640–2652, 10.1158/2767-9764.CRC-23-0218. 38047594 PMC10761659

[16] Y. Liu , Z. Su , O. Tavana , and W. Gu , “Understanding the Complexity of p53 in a New Era of Tumor Suppression,” Cancer Cell 42, no. 6 (2024): 946–967, 10.1016/j.ccell.2024.04.009. 38729160 PMC11190820

[17] S. M. Joruiz and J.‐C. Bourdon , “p53 Isoforms: Key Regulators of the Cell Fate Decision,” Cold Spring Harbor Perspectives in Medicine 6, no. 8 (2016): a026039, 10.1101/cshperspect.a026039. 26801896 PMC4968168

[18] F. Napoletano , B. Gibert , K. Yacobi‐Sharon , et al., “p53‐Dependent Programmed Necrosis Controls Germ Cell Homeostasis During Spermatogenesis,” PLoS Genetics 13, no. 9 (2017): 1007024, 10.1371/journal.pgen.1007024. PMC5629030 28945745

[19] S. M. Joruiz , J. A. Beck , I. Horikawa , and C. C. Harris , “The Δ133p53 Isoforms, Tuners of the p53 Pathway,” Cancers 12, no. 11 (2020): 3422, 10.3390/cancers12113422. 33218139 PMC7698932

[20] M.‐L. Dichtel‐Danjoy , D. Ma , P. Dourlen , et al., “Drosophila p53 Isoforms Differentially Regulate Apoptosis and Apoptosis‐Induced Proliferation,” Cell Death & Differentiation 20, no. 1 (2013): 108–116, 10.1038/cdd.2012.100. 22898807 PMC3524635

[21] M. Robin , A. R. Issa , C. C. Santos , et al., “Drosophila p53 Integrates the Antagonism Between Autophagy and Apoptosis in Response to Stress,” Autophagy 15, no. 5 (2019): 771–784, 10.1080/15548627.2018.1558001. 30563404 PMC6526837

[22] C. I. Wittke , E. C. Cheung , D. Athineos , et al., “p53 and TIGAR Promote Redox Control to Protect Against Metabolic Dysfunction‐Associated Steatohepatitis,” JHEP Reports 7, no. 7 (2025): 101397, 10.1016/j.jhepr.2025.101397. 40524696 PMC12167478

[23] L. Coronel , D. Häckes , K. Schwab , K. Riege , S. Hoffmann , and M. Fischer , “p53‐Mediated AKT and mTOR Inhibition Requires RFX7 and DDIT4 and Depends on Nutrient Abundance,” Oncogene 41, no. 7 (2022): 1063–1069, 10.1038/s41388-021-02147-z. 34907345 PMC8837532

[24] D. Khan , A. Katoch , A. Das , et al., “Reversible Induction of Translational Isoforms of p53 in Glucose Deprivation,” Cell Death & Differentiation 22, no. 7 (2015): 1203–1218, 10.1038/cdd.2014.220. 25721046 PMC4572868

[25] L. Q. Keoh , M. D. Ahmad Tarmizi , S. Sawai , L. Oh , and T. S. Ramasamy , “Short‐Term Hypoxia and Nutrient Deprivation Stress Induced Shifts in p53 Isoform Expression in HepG2 Tumourspheres,” Molecular Biology Reports 53, no. 1 (2026): 271, 10.1007/s11033-025-11415-0. 41518471

[26] K. Liu , Y. Zang , X. Guo , et al., “The Δ133p53 Isoform Reduces Wtp53‐Induced Stimulation of DNA Pol γ Activity in the Presence and Absence of D4T,” Aging and Disease 8, no. 2 (2017): 228–239, 10.14336/AD.2016.0910. 28400988 PMC5362181

[27] S.‐Y. Lee , Y. Jang , H.‐Y. Seok , and Y.‐H. Moon , “A Novel Mechanism of the p53 Isoform Δ40p53α in Regulating Collagen III Expression in TGFβ1 ‐Induced LX ‐2 Human Hepatic Stellate Cells,” FASEB Journal 39, no. 8 (2025): 70541, 10.1096/fj.202403146RR. PMC11999059 40232888

[28] S.‐Y. Lee , C. Robertson , A. Diot , V. Meuray , J.‐C. Bourdon , and M. J. Bissell , “Δ133p53 coordinates ECM‐Driven Morphogenesis and Gene Expression in Three‐Dimensional Mammary Epithelial Acini,” Journal of Cell Science 135, no. 21 (2022): jcs259673, 10.1242/jcs.259673. 36239052 PMC9687550

[29] F. Mantovani , L. Collavin , and G. Del Sal , “Mutant p53 as a Guardian of the Cancer Cell,” Cell Death & Differentiation 26, no. 2 (2019): 199–212, 10.1038/s41418-018-0246-9. 30538286 PMC6329812

[30] S. M. Joruiz , N. Von Muhlinen , I. Horikawa , M. R. Gilbert , and C. C. Harris , “Distinct Functions of Wild‐Type and R273H Mutant Δ133p53α Differentially Regulate Glioblastoma Aggressiveness and Therapy‐Induced Senescence,” Cell Death & Disease 15, no. 6 (2024): 454, 10.1038/s41419-024-06769-5. 38937431 PMC11211456

[31] D. Walerych , K. Lisek , R. Sommaggio , et al., “Proteasome Machinery Is Instrumental in a Common Gain‐of‐Function Program of the p53 Missense Mutants in Cancer,” Nature Cell Biology 18, no. 8 (2016): 897–909, 10.1038/ncb3380. 27347849

[32] E. Alvarado‐Ortiz , E. Ortiz‐Sánchez , M. A. Sarabia‐Sánchez , K. G. de la Cruz‐López , A. García‐Carrancá , and M. Robles‐Flores , “Mutant p53 Gain‐of‐Function Stimulates Canonical Wnt Signaling via PI3K/AKT Pathway in Colon Cancer,” Journal of Cell Communication and Signaling 17, no. 4 (2023): 1389–1403, 10.1007/s12079-023-00793-4. 37982965 PMC10713888

[33] M. Ghosh , S. Saha , J. Bettke , et al., “Mutant p53 Suppresses Innate Immune Signaling to Promote Tumorigenesis,” Cancer Cell 39, no. 4 (2021): 494–508.e5, 10.1016/j.ccell.2021.01.003. 33545063 PMC8044023

[34] G. Sorrentino , N. Ruggeri , V. Specchia , et al., “Metabolic Control of YAP and TAZ by the Mevalonate Pathway,” Nature Cell Biology 16, no. 4 (2014): 357–366, 10.1038/ncb2936. 24658687

[35] E. Ingallina , G. Sorrentino , R. Bertolio , et al., “Mechanical Cues Control Mutant p53 Stability Through a Mevalonate–RhoA Axis,” Nature Cell Biology 20, no. 1 (2018): 28–35, 10.1038/s41556-017-0009-8. 29255172 PMC6179142

[36] S.‐H. Moon , C.‐H. Huang , S. L. Houlihan , et al., “P53 Represses the Mevalonate Pathway to Mediate Tumor Suppression,” Cell 176, no. 3 (2019): 564–580.e19, 10.1016/j.cell.2018.11.011. 30580964 PMC6483089

[37] S. F. Brunner , I. Martincorena , G. Mannino , et al., “Somatic Genomics as a Discovery Engine for Biomedicine,” Cell 189, no. 5 (2026): 1269–1286, 10.1016/j.cell.2026.01.032.41795441

[38] L. Esteban‐Martínez and M. Torres , “Metabolic Regulation of Cell Competition,” Developmental Biology 475 (2021): 30–36, 10.1016/j.ydbio.2021.02.011. 33652024

[39] S. M. van Neerven and L. Vermeulen , “Cell Competition in Development, Homeostasis and Cancer,” Nature Reviews Molecular Cell Biology 24, no. 3 (2023): 221–236, 10.1038/s41580-022-00538-y. 36175766

[40] B. Salvador‐Barbero , M. Alatsatianos , J. P. Morton , O. J. Sansom , and C. Hogan , “KRASG12D Cells Override Homeostatic Cell Elimination Mechanisms in Adult Pancreas via Wnt5a and Cell Dormancy,” Gastroenterology 169, no. 5 (2025): 983–999.e21, 10.1053/j.gastro.2025.02.042.40204099

[41] H. Watanabe , K. Ishibashi , H. Mano , et al., “Mutant p53‐Expressing Cells Undergo Necroptosis via Cell Competition With the Neighboring Normal Epithelial Cells,” Cell Reports 23, no. 13 (2018): 3721–3729, 10.1016/j.celrep.2018.05.081. 29949757

[42] S. Kon , K. Ishibashi , H. Katoh , et al., “Cell Competition With Normal Epithelial Cells Promotes Apical Extrusion of Transformed Cells Through Metabolic Changes,” Nature Cell Biology 19, no. 5 (2017): 530–541, 10.1038/ncb3509. 28414314

[43] J. Lavalou , K. Kulakova , Y. J. Subramaniam , and E. Piddini , “Mechanisms of Cellular Fitness and Cell Competition: Towards an Integrated View,” Current Opinion in Cell Biology 96 (2025): 102571, 10.1016/j.ceb.2025.102571. 40784241 PMC7618938

[44] Y. J. Subramaniam and E. Piddini , “A Spotlight on Oxidative Metabolism in Oncogenic Transformation and Cell Competition,” Cancer Research 85, no. 16 (2025): 2961–2962, 10.1158/0008-5472.CAN-25-2374. 40479609

[45] K. Kozyrska , G. Pilia , M. Vishwakarma , et al., “p53 Directs Leader Cell Behavior, Migration, and Clearance During Epithelial Repair,” Science 375, no. 6581 (2022): abl8876, 10.1126/science.abl8876.35143293

[46] L. Wagstaff , M. Goschorska , K. Kozyrska , et al., “Mechanical Cell Competition Kills Cells via Induction of Lethal p53 Levels,” Nature Communications 7 (2016): 11373, 10.1038/ncomms11373. PMC4848481 27109213

[47] N. Kakiuchi and S. Ogawa , “Clonal Expansion in Non‐Cancer Tissues,” Nature Reviews Cancer 21, no. 4 (2021): 239–256, 10.1038/s41568-021-00335-3. 33627798

[48] X. Yu , Y. Zhang , S. Xiong , et al., “Omics Analyses of a Somatic Trp53R245W/+ Breast Cancer Model Identify Cooperating Driver Events Activating PI3K/AKT/mTOR Signaling,” Proceedings of the National Academy of Sciences 119 (2022): 2210618119, 10.1073/pnas.2210618119. PMC9659373 36322759

[49] J. M. McDaniel , R. L. Morrissey , D. Dibra , et al., “p53R172H and p53R245W Hotspot Mutations Drive Distinct Transcriptomes in Mouse Mammary Tumors Through a Convergent Transcriptional Mediator,” Cancer Research Communications 4 (2024): 1991–2007, 10.1158/2767-9764.CRC-24-0128. 38994678 PMC11310746

[50] D. Dibra , S. Xiong , S. M. Moyer , et al., “Mutant p53 Protects Triple‐Negative Breast Adenocarcinomas From Ferroptosis in Vivo,” Science Advances 10, no. 7 (2024): adk1835, 10.1126/sciadv.adk1835. PMC10866549 38354236

[51] Y. Zhang , S. Xiong , B. Liu , et al., “Somatic Trp53 Mutations Differentially Drive Breast Cancer and Evolution of Metastases,” Nature Communications 9, no. 1 (2018): 3953, 10.1038/s41467-018-06146-9. PMC6160420 30262850

[52] D. Dibra , S. M. Moyer , A. K. El‐Naggar , Y. Qi , X. Su , and G. Lozano , “Triple‐Negative Breast Tumors are Dependent on Mutant p53 for Growth and Survival,” Proceedings of the National Academy of Sciences 120, no. 34 (2023): 2308807120, 10.1073/pnas.2308807120. PMC10450424 37579145

[53] P. Yao , P. Xiao , Z. Huang , et al., “Protein‐Level Mutant p53 Reporters Identify Druggable Rare Precancerous Clones in Noncancerous Tissues,” Nature Cancer 4, no. 8 (2023): 1176–1192, 10.1038/s43018-023-00608-w. 37537298

[54] T. Bondar and R. Medzhitov , “P53‐Mediated Hematopoietic Stem and Progenitor Cell Competition,” Cell Stem Cell 6, no. 4 (2010): 309–322, 10.1016/j.stem.2010.03.002. 20362536 PMC2872065

[55] D. Fernandez‐Antoran , G. Piedrafita , K. Murai , et al., “Outcompeting p53‐Mutant Cells in the Normal Esophagus by Redox Manipulation,” Cell Stem Cell 25, no. 3 (2019): 329–341.e6, 10.1016/j.stem.2019.06.011. 31327664 PMC6739485

[56] R. L. Hanson and E. Batchelor , “Coordination of MAPK and p53 Dynamics in the Cellular Responses to DNA Damage and Oxidative Stress,” Molecular Systems Biology 18, no. 12 (2022): 11401, 10.15252/msb.202211401. PMC9724178 36472304

[57] T. J. Humpton and K. H. Vousden , “Regulation of Cellular Metabolism and Hypoxia by p53,” Cold Spring Harbor Perspectives in Medicine 6, no. 7 (2016): a026146, 10.1101/cshperspect.a026146. 27371670 PMC4930918

[58] Y. Ye , Z. Ou , C. Li , et al., “Mutant p53 Regulates Pyruvate Dehydrogenase Kinase 1 (PDK1) to Promote Proliferation and Migration in Breast Cancer,” Cancer Science 117, no. 2 (2026): 335–352, 10.1111/cas.70252. 41243575 PMC12861101

[59] M. Poles , R. Pacchiana , C. Mortali , et al., “Mutant p53 Affects the Mitochondrial Proteome, Promoting Mitochondrial Fragmentation and OXPHOS in Pancreatic Ductal Adenocarcinoma Cells,” FEBS Journal 293, no. 1 (2026): 156–174, 10.1111/febs.70223. 40819215

[60] A. N. B. D. Jesus , A. Taha , D. Wang , et al., “Increased Expression of the Δ133p53β Isoform Enhances Brain Metastasis,” International Journal of Molecular Sciences 24, no. 2 (2023): 1267, 10.3390/ijms24021267. 36674782 PMC9866425

[61] G. Gadea , N. Arsic , K. Fernandes , et al., “TP53 drives Invasion Through Expression of Its Δ133p53β Variant,” Elife 5 (2016): 14734, 10.7554/eLife.14734. PMC5067115 27630122

[62] K. Laue , S. Pozzi , J. Zerbib , et al., “p53 Inactivation Drives Breast Cancer Metastasis to the Brain Through SCD1 Upregulation and Increased Fatty Acid Metabolism,” Nature Genetics 58, no. 1 (2026): 116–131, 10.1038/s41588-025-02446-1. 41461910

[63] B. D. Giaimo , F. Ferrante , and T. Borggrefe , “Lysine and Arginine Methylation of Transcription Factors,” Cellular and Molecular Life Sciences 82, no. 1 (2024): 5, 10.1007/s00018-024-05531-6. 39680066 PMC11649617

[64] C. Tombari , A. Zannini , R. Bertolio , et al., “Mutant p53 Sustains Serine‐Glycine Synthesis and Essential Amino Acids Intake Promoting Breast Cancer Growth,” Nature Communications 14, no. 1 (2023): 6777, 10.1038/s41467-023-42458-1. PMC10600207 37880212

[65] R. Boidot , F. Végran , A. Meulle , et al., “Regulation of Monocarboxylate Transporter MCT1 Expression by p53 Mediates Inward and Outward Lactate Fluxes in Tumors,” Cancer Research 72, no. 4 (2012): 939–948, 10.1158/0008-5472.CAN-11-2474. 22184616

[66] H. A. Adissu , C. McKerlie , M. Di Grappa , et al., “Timp3 loss Accelerates Tumour Invasion and Increases Prostate Inflammation in a Mouse Model of Prostate Cancer,” Prostate 75, no. 16 (2015): 1831–1843, 10.1002/pros.23056. 26332574

[67] J. Beck , C. Turnquist , I. Horikawa , and C. Harris , “Targeting Cellular Senescence in Cancer and Aging: Roles of p53 and Its Isoforms,” Carcinogenesis 41, no. 8 (2020): 1017–1029, 10.1093/carcin/bgaa071. 32619002 PMC7422622

[68] K. Fujita , A. M. Mondal , I. Horikawa , et al., “p53 isoforms Δ133p53 and p53β Are Endogenous Regulators of Replicative Cellular Senescence,” Nature Cell Biology 11, no. 9 (2009): 1135–1142, 10.1038/ncb1928. 19701195 PMC2802853

[69] C. Heichler , K. Scheibe , A. Schmied , et al., “STAT3 Activation Through IL‐6/IL‐11 in Cancer‐Associated Fibroblasts Promotes Colorectal Tumour Development and Correlates With Poor Prognosis,” Gut 69, no. 7 (2020): 1269–1282, 10.1136/gutjnl-2019-319200. 31685519

[70] M. Ghosh , S. Saha , J. Li , D. C. Montrose , and L. A. Martinez , “P53 Engages the cGAS/STING Cytosolic DNA Sensing Pathway for Tumor Suppression,” Molecular Cell 83, no. 2 (2023): 266–280.e6, 10.1016/j.molcel.2022.12.023. 36638783 PMC9993620

[71] C. A. Ishak , S. A. Marhon , N. Tchrakian , et al., “Chronic Viral Mimicry Induction Following p53 Loss Promotes Immune Evasion,” Cancer Discovery 15, no. 4 (2025): 793–817, 10.1158/2159-8290.CD-24-0094. 39776167 PMC12776606

[72] F. Peng , J.‐H. Wang , W.‐J. Fan , et al., “Glycolysis Gatekeeper PDK1 Reprograms Breast Cancer Stem Cells Under Hypoxia,” Oncogene 37, no. 8 (2018): 1062–1074, 10.1038/onc.2017.368. 29106390 PMC5851116

[73] I. Horikawa , K.‐Y. Park , K. Isogaya , et al., “Δ133p53 Represses p53‐Inducible Senescence Genes and Enhances the Generation of Human Induced Pluripotent Stem Cells,” Cell Death & Differentiation 24, no. 6 (2017): 1017–1028, 10.1038/cdd.2017.48. 28362428 PMC5442472

[74] N. Arsic , G. Gadea , E. L. Lagerqvist , et al., “The p53 Isoform Δ133p53β Promotes Cancer Stem Cell Potential,” Stem Cell Reports 4, no. 4 (2015): 531–540, 10.1016/j.stemcr.2015.02.001. 25754205 PMC4400643

[75] L. Gong , X. Pan , H. Chen , et al., “P53 Isoform Δ133p53 Promotes Efficiency of Induced Pluripotent Stem Cells and Ensures Genomic Integrity During Reprogramming,” Scientific Reports 6 (2016): 37281, 10.1038/srep37281. 27874035 PMC5118801

[76] Y. Qu , J. Cui , Z. Wu , et al., “Mechano‐Regulation of Cancer Cell Memory in Tumor Progression and Therapy,” Mechanobiology in Medicine 4, no. 1 (2026): 100165, 10.1016/j.mbm.2025.100165. 41450974 PMC12731285

[77] C. T. Mierke , “The Matrix Environmental and Cell Mechanical Properties Regulate Cell Migration and Contribute to the Invasive Phenotype of Cancer Cells,” Reports on Progress in Physics 82, no. 6 (2019): 064602, 10.1088/1361-6633/ab1628. 30947151

[78] E. Piskounova , M. Agathocleous , M. M. Murphy , et al., “Oxidative Stress Inhibits Distant Metastasis by Human Melanoma Cells,” Nature 527, no. 7577 (2015): 186–191, 10.1038/nature15726. 26466563 PMC4644103

[79] H. Campbell , N. Fleming , I. Roth , et al., “∆133p53 Isoform Promotes Tumour Invasion and Metastasis via Interleukin‐6 Activation of JAK‐STAT and RhoA‐ROCK Signalling,” Nature Communications 9, no. 1 (2018): 254, 10.1038/s41467-017-02408-0. PMC5772473 29343721

[80] N. Light , M. Layeghifard , A. Attery , et al., “Germline TP53 Mutations Undergo Copy Number Gain Years Prior to Tumor Diagnosis,” Nature Communications 14, no. 1 (2023): 77, 10.1038/s41467-022-35727-y. PMC9816166 36604421

[81] V. Subasri , N. Light , N. Kanwar , et al., “Multiple Germline Events Contribute to Cancer Development in Patients With Li‐Fraumeni Syndrome,” Cancer Research Communications 3, no. 5 (2023): 738–754, 10.1158/2767-9764.CRC-22-0402. 37377903 PMC10150777

[82] R. Sajwan , L. Wang , O. Casar‐Borota , et al., “A Cancer‐Associated TP53 Synonymous Mutation Induces Synthesis of the p53 Isoform p53/47,” British Journal of Cancer 133, no. 7 (2025): 970–975, 10.1038/s41416-025-03127-w. 40715694 PMC12480914

[83] S. A. Schubert , D. Ruano , S. M. Joruiz , et al., “Germline Variant Affecting p53β Isoforms Predisposes to Familial Cancer,” Nature Communications 15, no. 1 (2024): 8208, 10.1038/s41467-024-52551-8. PMC11410958 39294166

[84] C. Clavería , G. Giovinazzo , R. Sierra , and M. Torres , “Myc‐driven Endogenous Cell Competition in the Early Mammalian Embryo,” Nature 500, no. 7460 (2013): 39–44, 10.1038/nature12389. 23842495

[85] R. Sancho , S. M. Blake , C. Tendeng , B. E. Clurman , J. Lewis , and A. Behrens , “Fbw7 Repression by hes5 Creates a Feedback Loop That Modulates Notch‐Mediated Intestinal and Neural Stem Cell Fate Decisions,” PLoS Biology 11, no. 6 (2013): 1001586, 10.1371/journal.pbio.1001586. PMC3679002 23776410

[86] C. Commisso , S. M. Davidson , R. G. Soydaner‐Azeloglu , et al., “Macropinocytosis of Protein is an Amino Acid Supply Route in Ras‐Transformed Cells,” Nature 497, no. 7451 (2013): 633–637, 10.1038/nature12138. 23665962 PMC3810415

[87] S. Papa , P. M. Choy , and C. Bubici , “The ERK and JNK Pathways in the Regulation of Metabolic Reprogramming,” Oncogene 38, no. 13 (2019): 2223–2240, 10.1038/s41388-018-0582-8. 30487597 PMC6398583

[88] A. Martin‐Vega and M. H. Cobb , “ERK1/2‐MAPK Signaling: Metabolic, Organellar, and Cytoskeletal Interactions,” Current Opinion in Cell Biology 95 (2025): 102526, 10.1016/j.ceb.2025.102526. 40344863

[89] M. Ziosi , L. A. Baena‐López , D. Grifoni , et al., “dMyc Functions Downstream of Yorkie to Promote the Supercompetitive Behavior of Hippo Pathway Mutant Cells,” PLoS Genetics 6, no. 9 (2010): 1001140, 10.1371/journal.pgen.1001140. PMC2944792 20885789

[90] C. de la Cova , N. Senoo‐Matsuda , M. Ziosi , et al., “Supercompetitor Status of Drosophila Myc Cells Requires p53 as a Fitness Sensor to Reprogram Metabolism and Promote Viability,” Cell Metabolism 19, no. 3 (2014): 470–483, 10.1016/j.cmet.2014.01.012. 24561262 PMC3970267

[91] H. Mamada , T. Sato , M. Ota , and H. Sasaki , “Cell Competition in Mouse NIH3T3 Embryonic Fibroblasts is Controlled by the Activity of Tead family Proteins and Myc,” Journal of Cell Science 128, no. 4 (2015): 790–803, 10.1242/jcs.163675. 25588835

[92] A. Panichewa and C. Lorthongpanich , “From Fitness to Fate: Hippo‐YAP‐mediated Cell Competition Influence on Stem Cells Activity,” Bioscience Reports 46, no. 2 (2026): BSR20253960, 10.1042/BSR20253960. 41604285 PMC12905497

[93] Y. Liu and W. Gu , “The Complexity of p53‐Mediated Metabolic Regulation in Tumor Suppression,” Seminars in Cancer Biology 85 (2022): 4–32, 10.1016/j.semcancer.2021.03.010. 33785447 PMC8473587

[94] X. Gu , Y. Zhu , J. Su , et al., “Lactate‐Induced Activation of Tumor‐Associated Fibroblasts and IL‐8‐Mediated Macrophage Recruitment Promote Lung Cancer Progression,” Redox Biology 74 (2024): 103209, 10.1016/j.redox.2024.103209. 38861833 PMC11215341

[95] X. Liu , H. Sun , J. Liang , et al., “Metabolic Interplay Between Endometrial Cancer and Tumor‐Associated Macrophages: Lactate‐Induced M2 Polarization Enhances Tumor Progression,” Journal of Translational Medicine 23, no. 1 (2025): 923, 10.1186/s12967-025-06235-6. 40826409 PMC12359828

[96] M. Kim , S. Hwang , B. Kim , et al., “YAP Governs Cellular Adaptation to Perturbation of Glutamine Metabolism by Regulating ATF4‐mediated Stress Response,” Oncogene 42, no. 38 (2023): 2828–2840, 10.1038/s41388-023-02811-6. 37591953

[97] L. Liang , W. Li , X. Li , et al., “‘Reverse Warburg Effect’ of Cancer‑Associated Fibroblasts (Review),” International Journal of Oncology 60, no. 6 (2022): 67, 10.3892/ijo.2022.5357. 35425996

[98] A. K. Witkiewicz , D. Whitaker‐Menezes , A. Dasgupta , et al., “Using the “Reverse Warburg Effect” to Identify High‐Risk Breast Cancer Patients: Stromal MCT4 Predicts Poor Clinical Outcome in Triple‐Negative Breast Cancers,” Cell Cycle 11, no. 6 (2012): 1108–1117, 10.4161/cc.11.6.19530. 22313602 PMC3335917

[99] C. Lefevre , M. M. Thibaut , A. Loumaye , et al., “Tumoral Acidosis Promotes Adipose Tissue Depletion by Fostering Adipocyte Lipolysis,” Molecular Metabolism 83 (2024): 101930, 10.1016/j.molmet.2024.101930. 38570069 PMC11027574

[100] Z. Cai , Y. Li , M. Ma , et al., “Adipocytes Promote Pancreatic Cancer Migration and Invasion Through Fatty Acid Metabolic Reprogramming,” Oncology Reports 50, no. 1 (2023): 141, 10.3892/or.2023.8578. 37264956 PMC10285608

[101] J. Gong , Y. Lin , H. Zhang , et al., “Reprogramming of Lipid Metabolism in Cancer‐Associated Fibroblasts Potentiates Migration of Colorectal Cancer Cells,” Cell Death & Disease 11, no. 4 (2020): 267, 10.1038/s41419-020-2434-z. 32327627 PMC7181758

[102] J. Ye , Y. Lu , W. Huang , et al., “The Lactylation‐Immunosuppression Network in Cancer: Driving a Metabolic‐Epigenetic Axis,” Frontiers in Immunology 17 (2026): 1752934, 10.3389/fimmu.2026.1752934. 41668750 PMC12884833

[103] H. C. Pruitt , Y. Guan , H. Liu , et al., “Collagen VI Deposition Mediates Stromal T Cell Trapping Through Inhibition of T Cell Motility in the Prostate Tumor Microenvironment,” Matrix Biology 121 (2023): 90–104, 10.1016/j.matbio.2023.06.002. 37331435 PMC13069691

[104] A. Bahrami , A. Khalaji , M. Bahri Najafi , et al., “NF‐κB Pathway and Angiogenesis: Insights Into Colorectal Cancer Development and Therapeutic Targets,” European Journal of Medical Research 29, no. 1 (2024): 610, 10.1186/s40001-024-02168-w. 39702532 PMC11658081

[105] M. Tufail , C.‐H. Jiang , and N. Li , “Altered Metabolism in Cancer: Insights Into Energy Pathways and Therapeutic Targets,” Molecular Cancer 23, no. 1 (2024): 203, 10.1186/s12943-024-02119-3. 39294640 PMC11409553

[106] K. Kane , D. Edwards , and J. Chen , “The Influence of Endothelial Metabolic Reprogramming on the Tumor Microenvironment,” Oncogene 44, no. 2 (2025): 51–63, 10.1038/s41388-024-03228-5. 39567756 PMC11706781

[107] A. Z. Ayob and T. S. Ramasamy , “Prolonged Hypoxia Switched on Cancer Stem Cell‐Like Plasticity in HepG2 Tumourspheres Cultured in Serum‐Free Media,” In Vitro Cellular & Developmental Biology—Animal 57, no. 9 (2021): 896–911, 10.1007/s11626-021-00625-y. 34750738

[108] K. H. Vousden and C. Prives , “Blinded by the Light: The Growing Complexity of p53,” Cell 137, no. 3 (2009): 413–431, 10.1016/j.cell.2009.04.037. 19410540

[109] S. Camus , S. Ménendez , K. Fernandes , et al., “The p53 Isoforms are Differentially Modified by Mdm2,” Cell Cycle 11, no. 8 (2012): 1646–1655, 10.4161/cc.20119. 22487680 PMC3341231

[110] D. A. Guertin and K. E. Wellen , “Acetyl‐CoA Metabolism in Cancer,” Nature Reviews Cancer 23, no. 3 (2023): 156–172, 10.1038/s41568-022-00543-5. 36658431 PMC11137663

[111] J.‐Y. Yin , X.‐T. Lu , M.‐L. Hou , T. Cao , and Z. Tian , “Sirtuin1‐p53: A Potential Axis for Cancer Therapy,” Biochemical Pharmacology 212 (2023): 115543, 10.1016/j.bcp.2023.115543. 37037265

[112] W. Sun , R. Liu , X. Gao , et al., “Targeting Serine‐Glycine‐One‐Carbon Metabolism as a Vulnerability in Cancers,” Biomarker Research 11, no. 1 (2023): 48, 10.1186/s40364-023-00487-4. 37147729 PMC10161514

[113] M. J. Gonzalez‐Rellan , M. F. Fondevila , U. Fernandez , et al., “O‐GlcNAcylated p53 in the Liver Modulates Hepatic Glucose Production,” Nature Communications 12, no. 1 (2021): 5068, 10.1038/s41467-021-25390-0. PMC8379189 34417460

[114] A. Indeglia , A. Valdespino , G. Pantella , et al., “Targeted Citrullination Enables p53 Binding to Non‐Canonical Sites,” Molecular Cell 85, no. 19 (2025): 3588–3604.e11, 10.1016/j.molcel.2025.09.004. 41043392 PMC12671568

[115] E. Panatta , A. Butera , E. Mammarella , et al., “Metabolic Regulation by p53 Prevents R‐Loop‐Associated Genomic Instability,” Cell Reports 41, no. 5 (2022): 111568, 10.1016/j.celrep.2022.111568. 36323249

[116] S. Kaypee , S. A. Sahadevan , S. Patil , et al., “Mutant and Wild‐Type Tumor Suppressor p53 Induces p300 Autoacetylation,” Iscience 4 (2018): 260–272, 10.1016/j.isci.2018.06.002. 30240745 PMC6147029

[117] R. Benedetti , M. Di Crosta , G. D'Orazi , and M. Cirone , “Post‐Translational Modifications (PTMs) of mutp53 and Epigenetic Changes Induced by mutp53,” Biology 13, no. 7 (2024): 508, 10.3390/biology13070508. 39056701 PMC11273943

[118] T. A. Grigoreva , A. A. Romanova , V. G. Tribulovich , et al., “p53: The Multifaceted Roles of Covalent Modifications in Cancer,” Pharmaceuticals 17, no. 12 (2024): 1682, 10.3390/ph17121682. 39770524 PMC11677429

[119] W. McKerrow , X. Wang , C. Mendez‐Dorantes , et al., “LINE‐1 Expression in Cancer Correlates With p53 Mutation, Copy Number Alteration, and S Phase Checkpoint,” Proceedings of the National Academy of Sciences 119, no. 8 (2022): 2115999119, 10.1073/pnas.2115999119. PMC8872788 35169076

[120] D. Ardeljan , J. P. Steranka , C. Liu , et al., “Cell Fitness Screens Reveal a Conflict Between LINE‐1 Retrotransposition and DNA Replication,” Nature Structural & Molecular Biology 27, no. 2 (2020): 168–178, 10.1038/s41594-020-0372-1. PMC7080318 32042151

[121] B. Pradhan , J. Oikkonen , K. Zhang , et al., “Dynamic and Ongoing De Novo L1 Retrotransposition Contributes to Genome Plasticity and Intrapatient Heterogeneity in Ovarian Cancer,” Cancer Research 86, no. 4 (2026): 1073–1088, 10.1158/0008-5472.CAN-24-4419. 41223332 PMC13055634

[122] N. Raj , M. Wang , J. A. Seoane , et al., “The Mettl3 Epitranscriptomic Writer Amplifies p53 Stress Responses,” Molecular Cell 82, no. 13 (2022): 2370–2384.e10, 10.1016/j.molcel.2022.04.010. 35512709 PMC9807187

[123] S. Sun , E. You , J. Hong , et al., “Cancer Cells Restrict Immunogenicity of Retrotransposon Expression via Distinct Mechanisms,” Immunity 57, no. 12 (2024): 2879–2894.e11, 10.1016/j.immuni.2024.10.015. 39577413 PMC12022969

[124] G. Mauri , L. Santorelli , F. Marasca , et al., “Early‐Onset Colorectal Cancers Exhibit Distinctive Placental‐Like Features,” Research Square (2025), 10.21203/rs.3.rs-7193450/v1.

[125] L. M. Perez , S. V. Venugopal , A. S. Martin , S. J. Freedland , D. Di Vizio , and M. R. Freeman , “Mechanisms Governing Lineage Plasticity and Metabolic Reprogramming in Cancer,” Trends in Cancer 10, no. 11 (2024): 1009–1022, 10.1016/j.trecan.2024.08.001. 39218770

[126] K. B. Chiappinelli and K. H. Burns , “Transposable Element Activation: A Hallmark of Cancer,” Cancer Discovery 16, no. 8 (2026): 1494–1509, 10.1158/2159-8290.CD-25-2164. 41997583 PMC13159020

[127] P. Wang , L.‐L. Chen , Y. Xiong , and D. Ye , “Metabolite Regulation of Epigenetics in Cancer,” Cell Reports 43, no. 10 (2024): 114815, 10.1016/j.celrep.2024.114815. 39368084

[128] Y. Liang , X. Qu , N. M. Shah , and T. Wang , “Towards Targeting Transposable Elements for Cancer Therapy,” Nature Reviews Cancer 24, no. 2 (2024): 123–140, 10.1038/s41568-023-00653-8. 38228901 PMC13127460

[129] O. Demaria , S. Cornen , M. Daëron , Y. Morel , R. Medzhitov , and E. Vivier , “Harnessing Innate Immunity in Cancer Therapy,” Nature 574, no. 7776 (2019): 45–56, 10.1038/s41586-019-1593-5. 31578484

[130] L. Schmidleithner , P. Stüve , and M. Feuerer , “Transposable Elements as Instructors of the Immune System,” Nature Reviews Immunology 25, no. 9 (2025): 696–706, 10.1038/s41577-025-01172-3. 40301669

[131] M. F. Grinwald , W. N. Saintilnord , and T. Wang , “Unmasked: Transposable Elements as Drivers and Targets in Cancer,” Trends in Genetics 42, no. 1 (2026): 30–45, 10.1016/j.tig.2025.08.002. 40935685

[132] Y. A. Arribas , B. Baudon , M. Rotival , et al., “Transposable Element Exonization Generates a Reservoir of Evolving and Functional Protein Isoforms,” Cell 187, no. 26 (2024): 7603–7620.e22, 10.1016/j.cell.2024.11.011. 39667937

[133] C. M. Laumont , K. Vincent , L. Hesnard , et al., “Noncoding Regions Are the Main Source of Targetable Tumor‐Specific Antigens,” Science Translational Medicine 10, no. 470 (2018): aau5516, 10.1126/scitranslmed.aau5516. 30518613

[134] S. Lheureux , C. Gourley , I. Vergote , and A. M. Oza , “Epithelial Ovarian Cancer,” Lancet 393, no. 10177 (2019): 1240–1253, 10.1016/S0140-6736(18)32552-2. 30910306

[135] A. V. Vylegzhanina , I. A. Bespalov , K. A. Novototskaya‐Vlasova , et al., “Cancer Relevance of Circulating Antibodies Against LINE‐1 Antigens in Humans,” Cancer Research Communications 3, no. 11 (2023): 2256–2267, 10.1158/2767-9764.CRC-23-0289. 37870410 PMC10631453

[136] C. Moschandrea and C. Frezza , “Metabolic Permissiveness: How Tissue Context Shapes Cancer,” Genes & Development 40 (2026): 287–307, 10.1101/gad.353516.125. 41663272 PMC12951767

[137] S. Sivanand , Y. Gultekin , P. S. Winter , et al., “Cancer Tissue of Origin Constrains the Growth and Metabolism of Metastases,” Nature Metabolism 6, no. 9 (2024): 1668–1681, 10.1038/s42255-024-01105-9. PMC11450831 39160333

[138] G. Bergers and S.‐M. Fendt , “The Metabolism of Cancer Cells During Metastasis,” Nature Reviews Cancer 21, no. 3 (2021): 162–180, 10.1038/s41568-020-00320-2. 33462499 PMC8733955

[139] K. Y. Koo , K. Moon , H. S. Song , and M.‐S. Lee , “Metabolic Regulation by p53: Implications for Cancer Therapy,” Molecules and Cells 48, no. 4 (2025): 100198, 10.1016/j.mocell.2025.100198. 39986611 PMC11925517

[140] Y. Peng‐Winkler , X.‐Z. Liu , S. M. L. Verheul , et al., “Steatosis Shapes Prognosis‐Defining Liver Metastasis Heterogeneity in CRC,” Nature (2026): 207–215, 10.1038/s41586-026-10686-2.42386979

[141] S. Drapela , B. M. Garcia , A. P. Gomes , and A. L. Correia , “Metabolic Landscape of Disseminated Cancer Dormancy,” Trends in Cancer 11, no. 4 (2025): 321–333, 10.1016/j.trecan.2024.10.005. 39510896 PMC11981868

[142] Y. Wang , L. Wang , Y. Wei , et al., “Advances in the Molecular Regulation Mechanism of Tumor Dormancy and Its Therapeutic Strategy,” Discover Oncology 15, no. 1 (2024): 184, 10.1007/s12672-024-01049-2. 38795254 PMC11127899

[143] S. Liu , X. Zhang , W. Wang , et al., “Metabolic Reprogramming and Therapeutic Resistance in Primary and Metastatic Breast Cancer,” Molecular Cancer 23, no. 1 (2024): 261, 10.1186/s12943-024-02165-x. 39574178 PMC11580516

[144] X. Meng , J. Z. Gao , S. M. T. Gomendoza , J. W. Li , and S. Yang , “Recent Advances of WEE1 Inhibitors and Statins in Cancers with p53 Mutations,” Frontiers in Medicine 8 (2021): 737951, 10.3389/fmed.2021.737951. 34671620 PMC8520942

[145] M. Pereira , K. Matuszewska , A. Glogova , and J. Petrik , “Mutant p53, the Mevalonate Pathway and the Tumor Microenvironment Regulate Tumor Response to Statin Therapy,” Cancers 14, no. 14 (2022): 3500, 10.3390/cancers14143500. 35884561 PMC9323637

[146] G. Zhou , J. Wang , M. Zhao , et al., “Gain‐of‐Function Mutant p53 Promotes Cell Growth and Cancer Cell Metabolism via Inhibition of AMPK Activation,” Molecular Cell 54, no. 6 (2014): 960–974, 10.1016/j.molcel.2014.04.024. 24857548 PMC4067806

[147] S. Halford , G. J. Veal , S. R. Wedge , et al., “A Phase I Dose‐Escalation Study of AZD3965, an Oral Monocarboxylate Transporter 1 Inhibitor, in Patients With Advanced Cancer,” Clinical Cancer Research 29, no. 8 (2023): 1429–1439, 10.1158/1078-0432.CCR-22-2263. 36652553 PMC7614436

[148] S. Verma , S. Budhu , I. Serganova , et al., “Pharmacologic LDH Inhibition Redirects Intratumoral Glucose Uptake and Improves Antitumor Immunity in Solid Tumor Models,” Journal of Clinical Investigation 134, no. 17 (2024): 177606, 10.1172/JCI177606. PMC11364391 39225102

[149] J. De Los Santos‐Jiménez , J. A. Campos‐Sandoval , F. J. Alonso , J. Márquez , and J. M. Matés , “GLS and GLS2 Glutaminase Isoenzymes in the Antioxidant System of Cancer Cells,” Antioxidants 13, no. 6 (2024): 745, 10.3390/antiox13060745. 38929183 PMC11200642

[150] J. Lee , Y. Seo , and J.‐L. Roh , “Emerging Therapeutic Strategies Targeting GPX4‐Mediated Ferroptosis in Head and Neck Cancer,” International Journal of Molecular Sciences 26, no. 13 (2025): 6452, 10.3390/ijms26136452. 40650229 PMC12250494

[151] Z. Seferbekova , A. Lomakin , L. R. Yates , and M. Gerstung , “Spatial Biology of Cancer Evolution,” Nature Reviews Genetics 24, no. 5 (2023): 295–313, 10.1038/s41576-022-00553-x. 36494509

[152] F. Zhang , Z. Zhou , P. Zhang , and S. Li , “Multi‐Omics Meets Premalignancy: Paving the Way for Early Prevention of Cancer,” Research 8 (2025): 0930, 10.34133/research.0930. 41255572 PMC12620627

